# Supplementary material for: Blocking interaction between SHP2 and PD‐1 denotes a novel opportunity for developing PD‐1 inhibitors
Source: EMBO Mol Med. 2020 May 11;12(6):e11571. doi: 10.15252/emmm.201911571 (PMC7278553; doi:10.15252/emmm.201911571)
Supplement: Supplementary file 1 — Appendix [file EMMM-12-e11571-s001.pdf]

Blocking interaction between SHP2 and PD-1 denotes a novel opportunity for developing PD-1 inhibitors

Zhenzhen Fan[1*], Yahui Tian[1*], Zhipeng Chen[1], Lu Liu[1], Qian Zhou[1], Jingjing He[2], James Coleman[3], Changjiang Dong[3], Nan Li[1], Junqi Huang[1], Chenqi Xu[4], Zhimin Zhang[5], Song Gao[2], Penghui Zhou[2#], Ke Ding[5#], Liang Chen[6,7#]

Appendix

Appendix Fig S1-S5 (Page 2-18)
Appendix Table S1 (Page 19-30): Exact P-values and statistical tests.
Appendix Table S2 (Page 31-32): Antibody dilution list.

Appendix Fig S1

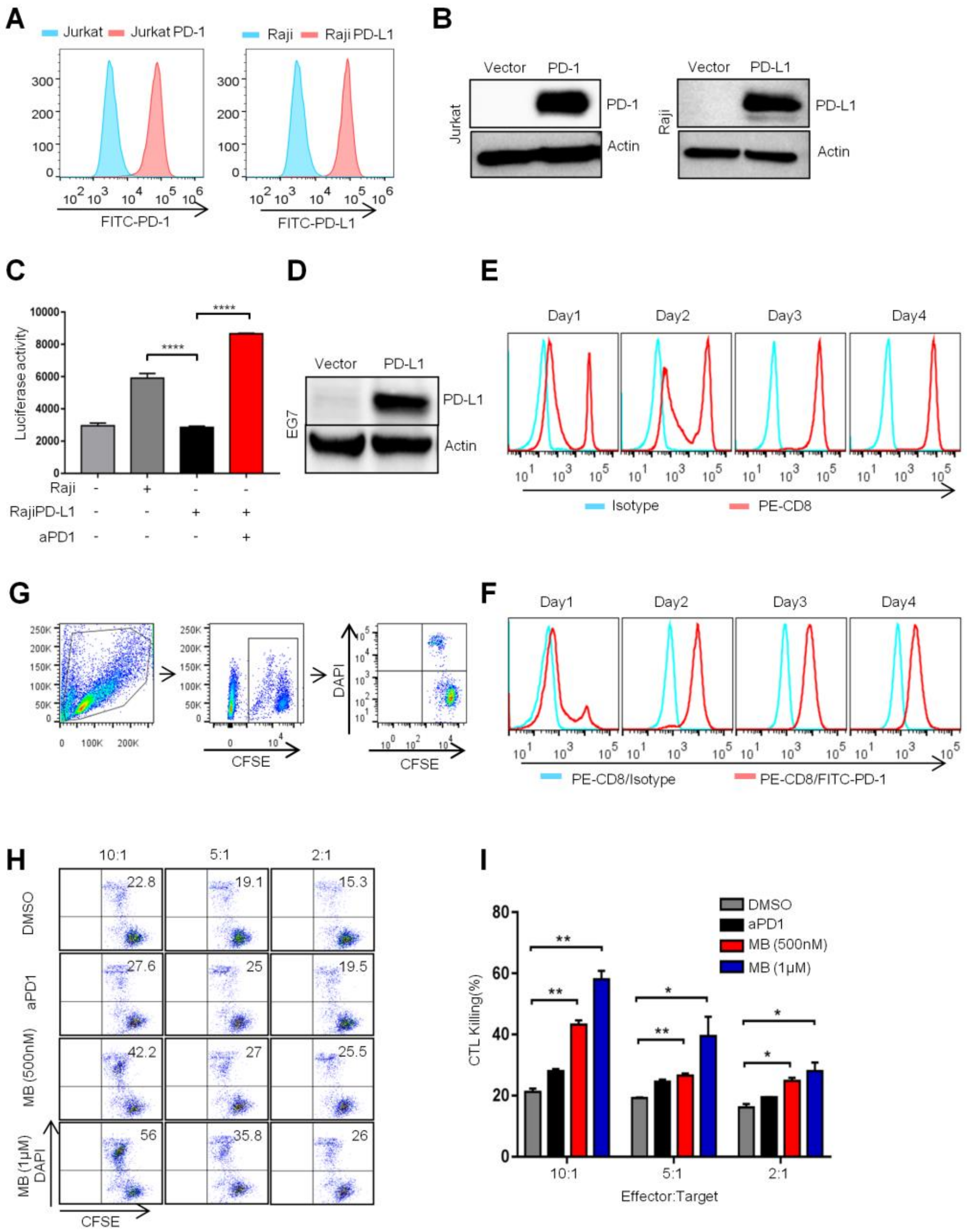

Appendix Fig S1

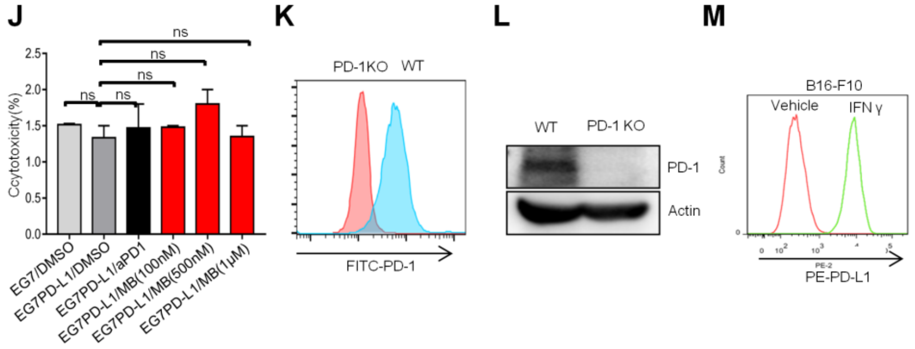

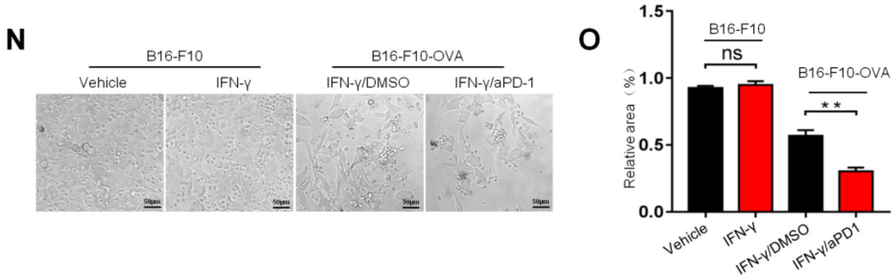

Appendix Fig S1. MB enhances cytotoxicity of activated CTL against PD-L1 expressing target cells

A. FACS histograms showing the expression of PD-1 in JP-luc stable cell line (left panel) and PD-L1 in Raji-L1 stable B cells (right panel). JP-luc: Jurkat cell harboring NFAT-luciferase transgene and overexpressing PD-1; Raji-L1: Raji overexpressing PD-L1.

B. Western blot analysis the expression of PD-1 in JP-luc stable cell line (left panel) and PD-L1 in Raji-L1 stable cell line (right panel).

C. Inhibition of PD1 resulted in activation of JP-luc cells. JP-luc cells were co-cultured with SEE-Loaded Raji or SEE-Loaded Raji-L1 cells for 6 hours in the presence of 10 μg/mL of PD-1 antibody.

D. Western blot analysis of PD-L1 expressing stable EG7 cell lines (referred to as EG7-L1).

E. FACS histograms analysis of CD8 population in splenocytes of OT-1 mice with media supplemented with 10 nM of SINFEEKL peptide on the indicated days after stimulation.

F. Daily FACS histograms analysis of expression of PD-1 in splenocytes of OT-1 mice with media supplemented with 10 nM of SINFEEKL peptide on the indicated days after stimulation.

G. FACS analysis method of (**EV1C**).

H. FACS analysis the cytotoxicity of OT-I CTLs against EG7-L1 in the presence of MB. aPD1 antibody served as positive control. Splenocytes from OT-I mice in culture were stimulated with 10 nM of SINFEEKL peptide for 3 days to generate mature CTLs. CTLs were incubated with CFSE labeled EG7-L1 cells in the presence of MB at indicated concentrations. Cytotoxicity was determined by flow cytometry. Data are representative of three independent

experiments. (effector to target ratio = 10:1, 5:1, 2:1, unpaired t-test. killing time:4h). EG7-L1: EG7 overexpressing PD-L1.

I. Statistical results of (**H**).

J. The Cytotoxicity of MB on EG7 or EG7-L1.

K. FACS analysis the expression of PD-1 in CTL from PD-1$^{-/-}$ (PD-1KO) mice or WT mice.

L. Western blot analysis the expression of PD-1 in CTL from PD-1KO mice or WT mice.

M. FACS histograms analysis of expression of PD-L1 in IFNγ treated B16-F10 cells.

N. PD-1 antibody enhanced Cytotoxicity of OT-I CTLs against IFNγ-treated B16-F10-OVA (scale bar = 50μm).

O. Statistical results of (**N**).

Data information: Data are representative of three independent experiments, and were analyzed by unpaired t-test. Error bars denote s.e.m. *P < 0.05; **P < 0.01; ****P < 0.0001.

Source data are available online for this figure.

Appendix Fig S2

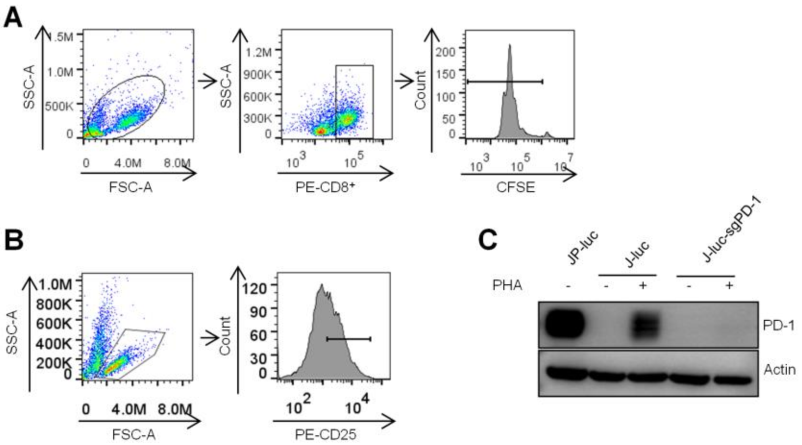

Appendix Fig S2. MB enhanced activation and effector function of CTL

A. FACS analysis of proliferation of OT-I CTLs. CTLs were labeled with 5 nM of CFSE. Dilution of CFSE were shown as histograms on $CD8^+$ T cells populations.

B. FACS analysis the surface expression of CD25.

C. Western blot analysis of PD-1 expression in JP-luc, J-luc and J-luc-sgPD-1 stable cell line stimulate without or with 500 ng/mL of PHA for 2 days. J-luc: Jurkat cell harboring NFAT-luciferase transgene; JP-luc: J-luc cell overexpressing PD-1; J-luc-sgPD-1: J-luc cells treated with lentivirus expressing sgPD-1/CAS9 simultaneously.

Source data are available online for this figure.

Appendix Fig S3

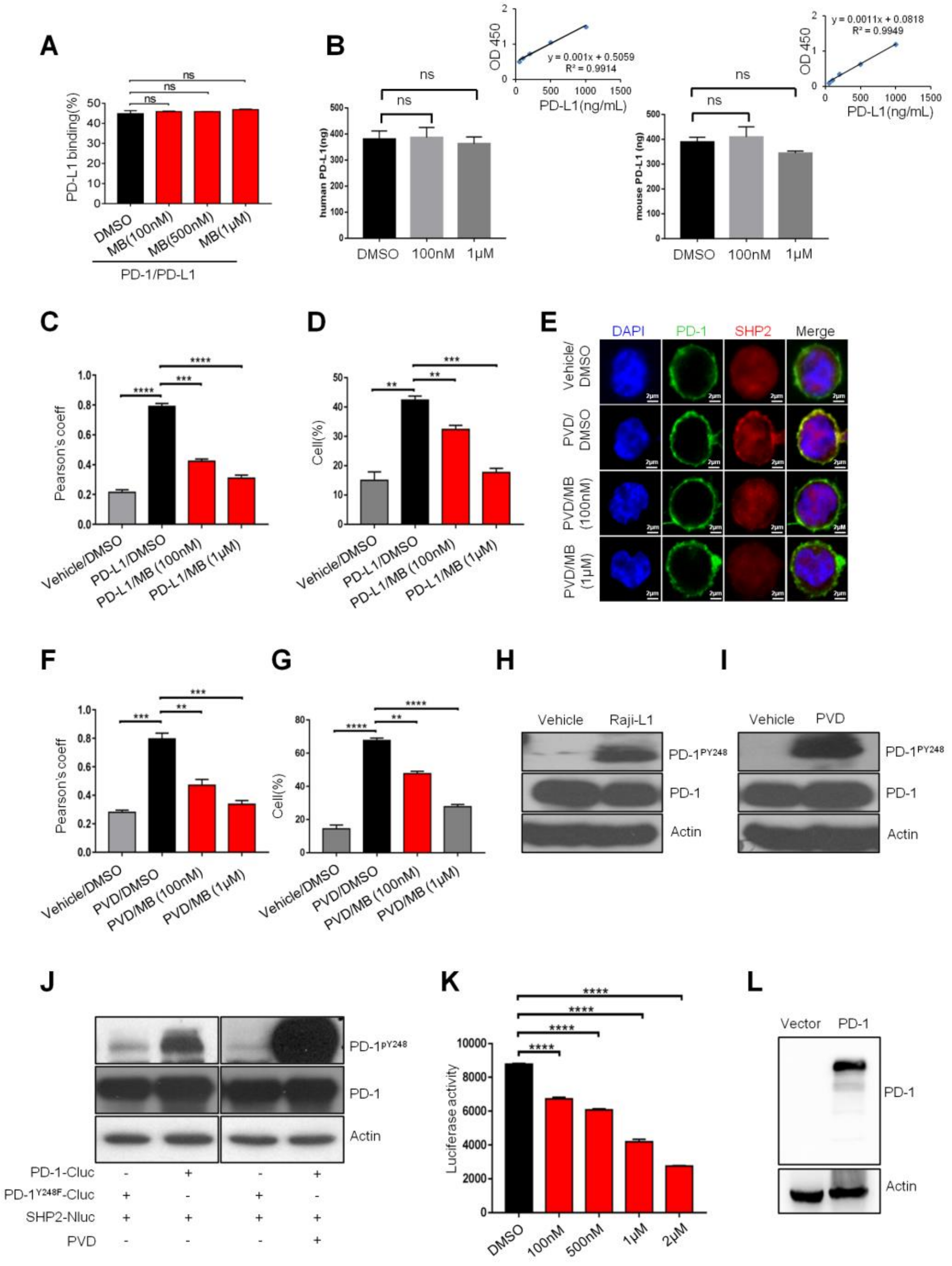

Appendix Fig S3

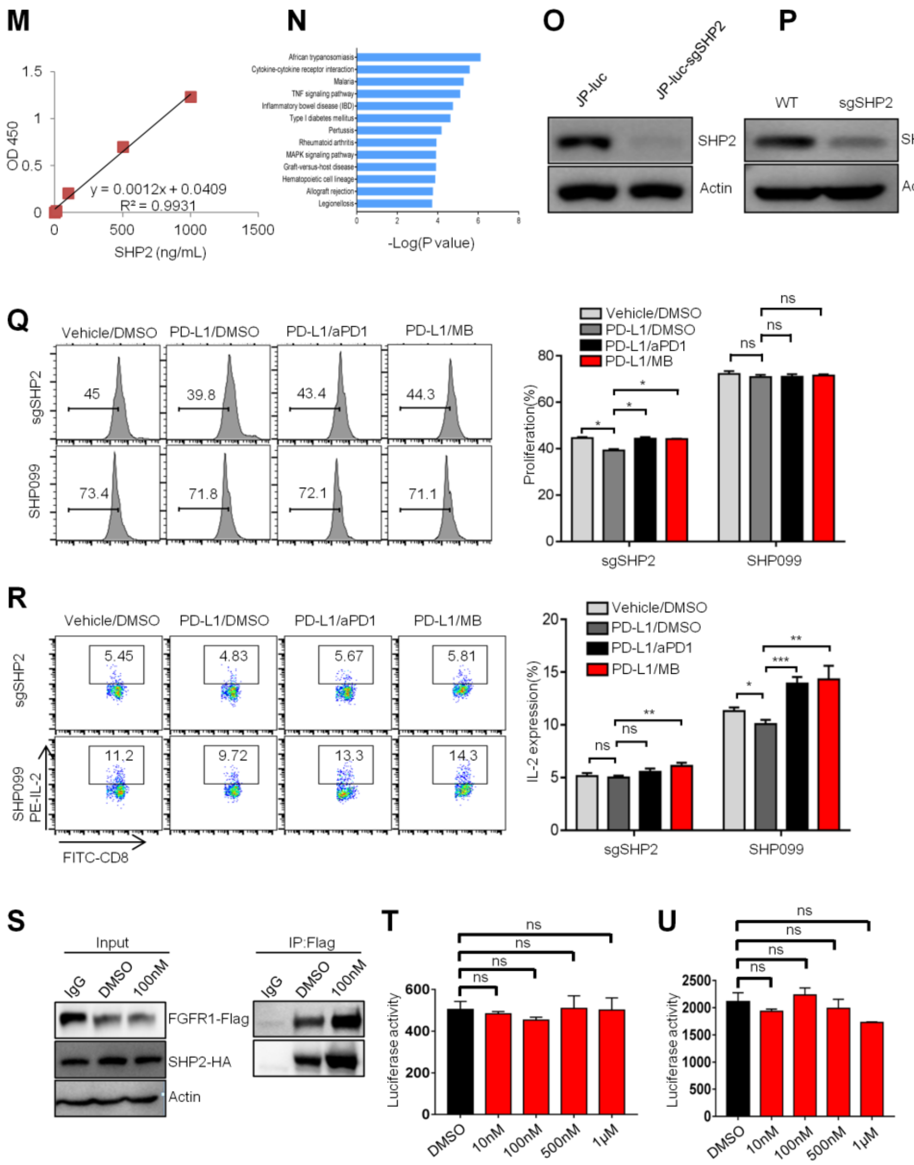

Appendix Fig S3. MB suppress PD-1 signaling through blocking SHP2 recruitment by PD-1

A. Bar graph of (EV3A).

B. Impact of MB on the interaction between PD-1 and PD-L1 protein revealed through ELISA analysis. 10 µg/mL of PD-1 protein coated on 96-well plate overnight. PD-L1-Fc fusion protein were incubated for 1 hour at RT. Plate-bound PD-L1 were then measured with horse-radish peroxidase conjugated anti-Fc secondary antibody.

C. Colocalization signals of PD-1-EGFP/SHP2-mCherry were quantitatively analyzed with LAS AF Lite software and displayed as Pearson's coefficient. Five horizons were randomly selected at each time (Pearson's coefficient between 0 and 1. 0 denotes no colocalization; 1 denotes complete colocalization).

D. Statistics of Jurkat cells showing colocalization of EGFP/mCherry. 4 fields were randomly chosen under by confocal microscope and percentage of cells showing colocalization of EGFP/mCherry were shown as mean value.

E. Jurkat cells showing EGFP-mCherry colocalization under confocal microscope analysis. Jurkat cells co-expressing PD-1-EGFP and SHP2-mCherry were incubated with MB for 1 hour with the media supplemented with 2 µM of PVD for the last 5min. Cells were then fixed with 4% Paraformaldehyde and stained with DAPI. (scale bar = 2 µm).

F. Pearson's coefficient determined in (**E**).

G. Percentage of Jurkat cells showing EGFP-mCherry colocalization were calculated against total cell population in (**E**).

H. Representative western blot showing the levels PD-1 pY248 phosphorylation from the lysates of the indicated Jurkat-PD-1 cells. Jurkat-PD-1 cells were cocultured with parental Raji cells

or Raji-L1. Cell lysate were subject to SDS-PAGE and total PD-1 and Y248 phosphorylated PD-1 were analyzed through western blot.

I. Representative western blot showing the levels PD-1 pY248 phosphorylation from the lysates of the indicated Jurkat-PD-1 cells. Jurkat-PD-1 cells were cocultured with PVD. Cell lysate were subject to SDS-PAGE and total PD-1 and Y248 phosphorylated PD-1 were analyzed through western blot.

J. Representative western blot showing the levels PD-1 pY248 phosphorylation from the lysates of the indicated 293T cells. Stable 293T cells clones stably co-expressing PD-1-C-luc or PD-1 Y248F-C-luc and SHP2-N-luc, treated w/o PVD for 5 minutes.

K. Luciferase complementation analysis of the effect of MB on the interaction between PD-1 with SHP2. 293T cells stably co-expressing PD-1-C-luc and SHP2-N-luc were treated with MB at indicated concentration for 6 hours in the presence of PVD for the last 5 minutes. Luciferase activity was measured as readout for PD-1/SHP2 interaction.

L. PD-1 protein expression in 293T. 293T cells were transfect with PD-1 expressing plasmid or control vector for 48h. Cell lysate were subject to SDS-PAGE for western blot analysis with anti-PD-1 antibody.

M. Standard Curve of figure **3D**.

N. Alteration of signaling pathways of OT-1 CTL at transcriptomic level when treated with MB. OT-1 CTLS were treated with 10 ng/mL of mouse PD-L1 protein and w/o 100nM MB for 24 hours. mRNAs were harvested for RNA-sequencing. Genes upregulated in MB treated cells were analyzed by KEGG. Top 13 pathways were graphed based on -log value of statistical significance.

O. Western blot analysis of SHP2 expression in JP-luc and JP-luc-sgSHP2. JP-luc: J-luc cell overexpressing PD-1; J-luc-sgPD-1: J-luc cells treated with lentivirus expressing sgSHP2/CAS9 simultaneously.

P. Western blot analysis of SHP2 expression in CTL infected with sgSHP2/CAS9 lentivirus or control (WT) cells.

Q. Effect of MB on the proliferation of sgSHP2 CTLs and 10 μM of SHP2099.

R. FACS analysis of IL-2 expression by CTLs of indicated genetic backgrounds and treatment.

S. Co-IP analysis of impact of MB on interaction between SHP2 and FGFR1.

T. Fluorescence complementation analysis of the effect of MB on the interaction between SHP2 and EGFR.

U. Fluorescence complementation analysis of the effect of MB on the interaction between SHP2 and FGFR1.

Data information: Data are representative of three independent experiments, and were analyzed by unpaired t-test. **$P < 0.01$; ***$P < 0.001$; ****$P < 0.0001$.

Source data are available online for this figure.

Appendix Fig S4

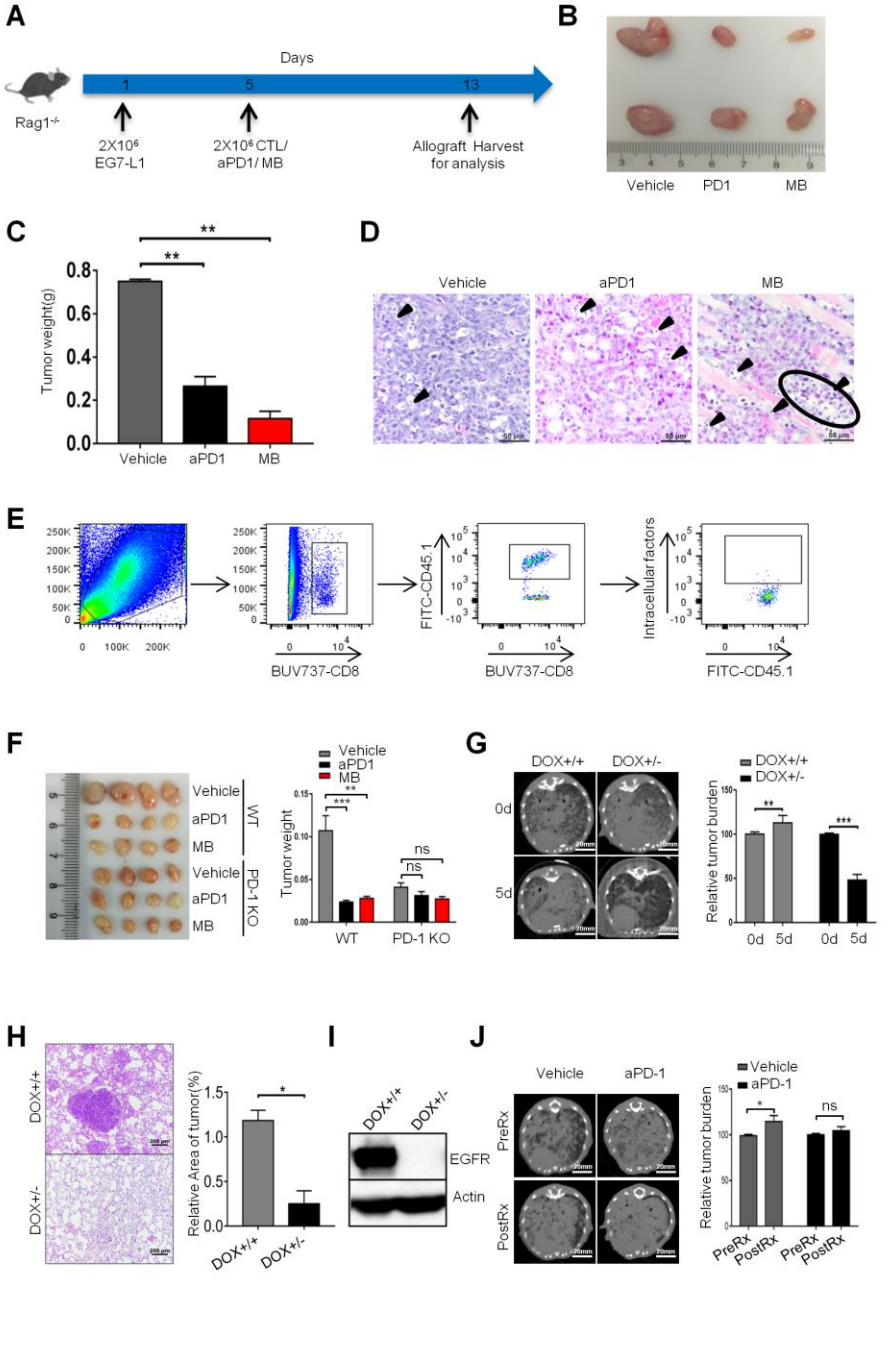

Appendix Fig S4

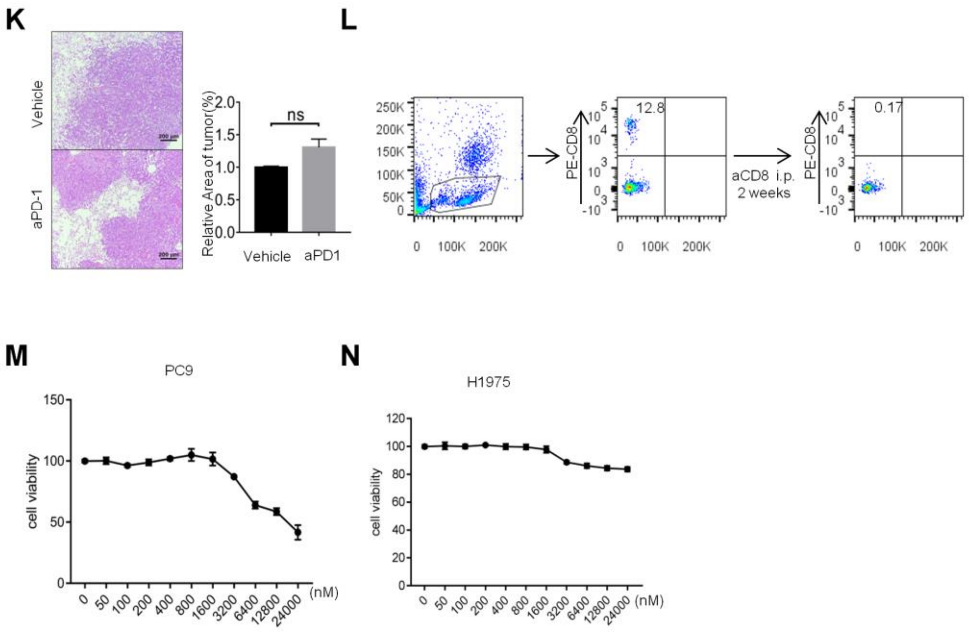

Appendix Fig S4. MB shrinks tumor *in vivo* through enhancing cytotoxic function of CTL

A. Schematic of the allograft mouse model for MB treatment. EG7-L1 cells were inoculated (s.c.) into the right flank of Rag1$^{-/-}$ mice on day 1 and OT1 CTLs were injected (i.v.) on day 5. Mice were randomized into 3 groups (n=2) and treated with vehicle, aPD1 (10 mg/kg, every other day, i.p.) or MB (i.g. 20mg/kg/day). Tumor size was measured by calipering every other day. EG7-L1: EG7 overexpressing PD-L1.

B. Representative image of the tumors on day 13 in (A).

C. Tumor weight on day 13 (n = 2) in (A).

D. Haematoxylin and Eosin staining for figure 4C. Apoptotic bodies are pointed with triangle. The area of dense apoptotic bodies in MB treated tumors are highlighted with solid circle.

E. FACS analysis of the expression of effector molecules by CD8^{+} CD45.1^{+} tumor-infiltrating lymphocytes (TILs).

F. Image of the tumors and bar graph of tumor weight (n=4).

G. Characterization of TetO-**E**GFR L858R/**C**C10rtTA mice (referred to as EC mice). EC mice (n = 3) were fed with doxycycline diet for 2 weeks and tumor burdens were recorded through Computed tomography scanning. The mice were fed with normal diet (withdrawing Doxycycline) for 5 days and CT imaged again. Left panel showing lung tumors continued growing in the mice fed with doxycycline diet (DOX$^{+/+}$). Right panel showing the lung tumor regression when withdrawing doxycycline for 5 days (DOX$^{+/-}$).

H. Haematoxylin and Eosin staining (lung section = 3) for (**G**).

I. Western Blot analysis of EGFR expression in lung of mice shown in (**G**).

J. PD-1 antibody treatment is ineffective to shrink EC tumors (n=3). Computed tomography for

EC mice were shown before and after treatment with aPD1 antibody.

K. Haematoxylin and Eosin staining result of lung section of EC mice (n = 3, lung section = 3) treated with aPD1 antibody.

L. Depleting $CD8^+$ T cells in EC mice. EC mice were treated with aCD8 antibody (200 μg per mouse, every other day, i.p.) to deplete $CD8^+$ T cells. Peripheral $CD8^+$ T cells were monitored through FACS analysis. Result indicated the $CD8^+$ T cells were almost completely depleted after treatment with aCD8 antibody for 2 weeks.

M. Effects of MB on cell viability. PC9 cells were treated with MB at indicated concentrations for 48 h. CCK-8 assay was performed to measure cell viability.

N. Effects of MB on cell viability. H1975 cells were treated with MB at indicated concentrations for 48 h. CCK-8 assay was performed to measure cell viability.

Data information: Data are representative of three independent experiments, and were analyzed by unpaired t-test. Error bars denote s.e.m. *$P < 0.05$; **$P < 0.01$; ***$P < 0.001$.

Source data are available online for this figure.

Appendix Fig S5

A

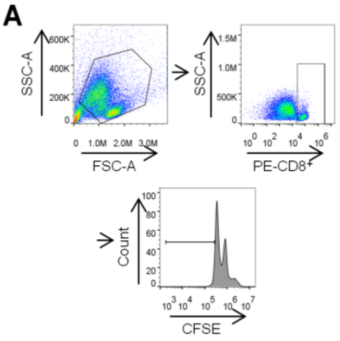

Appendix Fig S5. MB is effective to activate human $CD8^+$ T cells

A. CFSE dilution of CD8+ T cells through FACS analysis. CFSE (5nM) labeled human peripheral blood mononuclear cells (PBMC) were pre-incubated with DMSO, MB, 25 µg/ml of pembrolizumab or 20 µg/ml of Nivolumab for 1 h and then seeded in the 96-well plate precoated with 10 µg/ml of aCD3/aCD28 in the presence of 10 µg/ml human PD-L1 Protein. Cell proliferation was analyzed through FACS analysis, Dilution of CFSE are shown as histograms on CD8+ T cells populations.

Appendix Table S1: Exact P-values and statistical tests

Fig1C	P-value	Test
EG7/DMSO vs EG7PD-L1/DMSO	<0.0001	unpaired t-test
EG7PD-L1/DMSO vs EG7PD-L1/aPD1	<0.0001	unpaired t-test
EG7PD-L1/DMSO vs EG7PD-L1/100nM	0.0010	unpaired t-test
EG7PD-L1/DMSO vs EG7PD-L1/500nM	0.0005	unpaired t-test
EG7PD-L1/DMSO vs EG7PD-L1/1μM	0.0004	unpaired t-test

Fig1D		P-value	Test
10:1	DMSO vs aPD1	0.0039	unpaired t-test
	DMSO vs MB	0.0017	unpaired t-test
5:1	DMSO vs aPD1	0.0006	unpaired t-test
	DMSO vs MB	0.0001	unpaired t-test
2:1	DMSO vs aPD1	0.0007	unpaired t-test
	DMSO vs MB	0.0004	unpaired t-test

Fig1F	P-value	Test
EG7/DMSO vs EG7PD-L1/DMSO	0.1150	unpaired t-test
EG7PD-L1/DMSO vs EG7PD-L1/aPD1	0.3060	unpaired t-test
EG7PD-L1/DMSO vs EG7PD-L1/MB	0.4319	unpaired t-test

Fig1H	P-value	Test
DMSO vs MB	0.1483	unpaired t-test
IFN-γ+DMSO vs IFN-γ+aPD1	<0.0001	unpaired t-test
IFN-γ+DMSO vs IFN-γ+MB	<0.0001	unpaired t-test

Fig2B		P-value	Test
WT	CON/DMSO vs PD-L1/DMSO	<0.0001	unpaired t-test
	PD-L1/DMSO vs PD-L1/aPD1	<0.0001	unpaired t-test
	PD-L1/DMSO vs PD-L1/MB	<0.0001	unpaired t-test

PD-1 KO	PD-L1/DMSO vs PD-L1/DMSO	0.7110	unpaired t-test
	PD-L1/DMSO vs PD-L1/aPD1	0.5461	unpaired t-test
	PD-L1/DMSO vs PD-L1/MB	0.5543	unpaired t-test

Fig2D		P-value	Test
CD25	CON/DMSO vs PD-L1/DMSO	<0.0001	unpaired t-test
	PD-L1/DMSO vs PD-L1/aPD1	<0.0001	unpaired t-test
	PD-L1/DMSO vs PD-L1/MB	<0.0001	unpaired t-test
CD69	PD-L1/DMSO vs PD-L1/DMSO	0.0007	unpaired t-test
	PD-L1/DMSO vs PD-L1/aPD1	<0.0001	unpaired t-test
	PD-L1/DMSO vs PD-L1/MB	<0.0001	unpaired t-test

Fig2F		P-value	Test
JP-luc	Raji vs Raji PD-L1	0.0012	unpaired t-test
	Raji PD-L1 vs Raji PD-L1+aPD1	0.0045	unpaired t-test
	Raji PD-L1 vs Raji PD-L1+MB	0.0017	unpaired t-test
J-luc-sgPD-1	Raji vs Raji PD-L1	0.1776	unpaired t-test
	Raji PD-L1 vs Raji PD-L1+aPD1	0.3206	unpaired t-test
	Raji PD-L1 vs Raji PD-L1+MB	0.5050	unpaired t-test

Fig2G	P-value	Test
DMSO vs 100nM	0.0093	unpaired t-test
DMSO vs 1μM	0.0027	unpaired t-test

Fig2H	P-value	Test
Raji vs Raji PD-L1	0.0049	unpaired t-test
Raji PD-L1 vs Raji PD-L1+aPD1	0.0089	unpaired t-test
Raji PD-L1 vs Raji PD-L1+MB	<0.0001	unpaired t-test

Fig2J		P-value	Test
PE-IL-2	DMSO vs aPD1	0.0240	unpaired t-test
	DMSO vs 100nM	0.0034	unpaired t-test
	DMSO vs 1μM	0.0018	unpaired t-test
APC-Perforin	DMSO vs aPD1	0.0019	unpaired t-test
	DMSO vs 100nM	0.0011	unpaired t-test
	DMSO vs 1μM	0.0004	unpaired t-test

Fig3B		P-value	Test
Resting	PD-1^{Y248}-Cluc vs PD-1-Cluc	0.0016	unpaired t-test
Active/DMSO	PD-1^{Y248}-Cluc vs PD-1-Cluc	<0.0001	unpaired t-test
Active/PD-L1	PD-1^{Y248}-Cluc vs PD-1-Cluc	<0.0001	unpaired t-test
Active/PD-L1/MB	PD-1^{Y248}-Cluc vs PD-1-Cluc	0.0413	unpaired t-test
	Active/PD-L1vs Active/PD-L1/MB	<0.0001	unpaired t-test

Fig3D		P-value	Test
SHP2-FC Binding	DMSO vs 100nM MB	0.04909	unpaired t-test
	DMSO vs 500nM MB	0.0209	unpaired t-test
	DMSO vs 1μM MB	0.0096	unpaired t-test

Fig3H		P-value	Test
CD28-Cluc	Jurkat vs Jurkat PD-1	<0.0001	unpaired t-test
CD28^{Y191F}-Cluc	CD28-Cluc vs CD28^{Y191F}-Cluc	<0.0001	unpaired t-test
PD-L1	CD28-Cluc vs PD-L1	<0.0001	unpaired t-test
MB	PD-L1 vs v PD-L1 MB	<0.0001	unpaired t-test

Fig4B	Days after tumor injection	P-value	Test
Vehicle vs aPD1	7	0.0061	unpaired t-test
	10	0.0001	unpaired t-test
Vehicle vs MB	7	0.0476	unpaired t-test
	10	0.009	unpaired t-test

Fig4D	P-value	Test
Vehicle vs aPD1	0.0009	unpaired t-test
Vehicle vs MB	0.0010	unpaired t-test

Fig4F		P-value	Test
CD8	Vehicle vs aPD1	0.0411	unpaired t-test
	Vehicle vs MB	0.0020	unpaired t-test
Ki-67	Vehicle vs aPD1	0.0115	unpaired t-test
	Vehicle vs MB	0.0011	unpaired t-test

Fig4H		P-value	Test
Perforin	Vehicle vs aPD1	0.0227	unpaired t-test
	Vehicle vs MB	0.0025	unpaired t-test
GZMB	Vehicle vs aPD1	0.0208	unpaired t-test
	Vehicle vs MB	0.0050	unpaired t-test
IL-2	Vehicle vs aPD1	0.0063	unpaired t-test
	Vehicle vs MB	0.0043	unpaired t-test

Fig4K		P-value	Test
Vehicle	PreRx vs PostRx	0.0073	unpaired t-test
MB	PreRx vs PostRx	0.0001	unpaired t-test
aCD8/MB	PreRx vs PostRx	0.0065	unpaired t-test

Fig4M	P-value	Test
Vehicle vs MB	<0.0001	unpaired t-test
Vehicle vs aCD8/MB	0.0572	unpaired t-test

Fig5C	P-value	Test
Raji vs Raji PD-L1	<0.0001	unpaired t-test
Raji PD-L1 vs<br>Raji PD-L1+ Niv	0.0011	unpaired t-test
Raji PD-L1 vs<br>Raji PD-L1+ Pem	0.0024	unpaired t-test
Raji PD-L1 vs<br>Raji PD-L1+0.1μM	0.0022	unpaired t-test
Raji PD-L1 vs<br>Raji PD-L1+0.5μM	0.0012	unpaired t-test

Raji PD-L1+1μM	0.0013	unpaired t-test

Fig EV1D	P-value	Test
Raji vs Raji PD-L1	<0.0001	unpaired t-test
Raji PD-L1 vs Raji PD-L1+ aPD1	<0.0001	unpaired t-test

Fig EV1K		P-value	Test
10:1	EG7/Vehicle vs EG7PD-L1/Vehicle	0.0001	unpaired t-test
	EG7PD-L1/Vehicle vs EG7PD-L1/aPD1	0.0004	unpaired t-test
5:1	EG7/Vehicle vs EG7PD-L1/Vehicle	<0.0001	unpaired t-test
	EG7PD-L1/Vehicle vs EG7PD-L1/aPD1	0.0002	unpaired t-test
2:1	EG7/Vehicle vs EG7PD-L1/Vehicle	<0.0001	unpaired t-test
	EG7PD-L1/Vehicle vs EG7PD-L1/aPD1	0.0001	unpaired t-test

Fig EV1M		P-value	Test
10:1	DMSO vs 500nM MB	0.0032	unpaired t-test
	DMSO vs 1μM MB	0.0034	unpaired t-test
5:1	DMSO vs 500nM MB	0.0048	unpaired t-test
	DMSO vs 1μM MB	0.0458	unpaired t-test
2:1	DMSO vs 500nM MB	0.0157	unpaired t-test
	DMSO vs 1μM MB	0.0312	unpaired t-test

Fig EV1N	P-value	Test
EG7/DMSO vs EG7PD-L1/DMSO	0.3908	unpaired t-test
EG7PD-L1/DMSO vs EG7PD-L1/aPD1	0.7495	unpaired t-test
EG7PD-L1/DMSO vs EG7PD-L1/100nM	0.4750	unpaired t-test
EG7PD-L1/DMSO vs EG7PD-L1/500nM	0.2148	unpaired t-test
EG7PD-L1/DMSO vs EG7PD-L1/1μM	0.9525	unpaired t-test

Fig EV1S	P-value	Test
CON vs IFN-γ	0.5345	unpaired t-test

IFN-γ/DMSO vs IFN-γ/aPD1	0.0071	unpaired t-test

Fig EV1U	P-value	Test
DMSO vs 100nM	0.0455	unpaired t-test
DMSO vs 500nM	0.0426	unpaired t-test
DMSO vs 1µM	0.0078	unpaired t-test

Fig EV2B		P-value	Test
24h	PD-L1/Vehicle vs CON/Vehicle	0.2929	unpaired t-test
	PD-L1/Vehicle vs PD-L1/aPD1	0.0389	unpaired t-test
48h	PD-L1/Vehicle vs CON/Vehicle	0.0229	unpaired t-test
	PD-L1/Vehicle vs PD-L1/aPD1	0.0005	unpaired t-test
72h	PD-L1/Vehicle vs CON/Vehicle	0.0369	unpaired t-test
	PD-L1/Vehicle vs PD-L1/aPD1	0.0007	unpaired t-test

Fig EV2G	P-value	Test
DMSO vs aPD1	0.0042	unpaired t-test
DMSO vs 100nM	0.0220	unpaired t-test
DMSO vs 1µM	0.0012	unpaired t-test

Fig EV2H	P-value	Test
DMSO vs aPD1	0.0398	unpaired t-test
DMSO vs 100nM	0.0216	unpaired t-test
DMSO vs 1µM	0.0083	unpaired t-test

Fig EV3B	P-value	Test
DMSO vs 100nM	0.5869	unpaired t-test
DMSO vs 500nM	0.5805	unpaired t-test
DMSO vs 1µM	0.3185	unpaired t-test

Fig EV3C		P-value	Test
Human PD-L1	DMSO vs 100nM	0.9005	unpaired t-test
	DMSO vs 1µM	0.7643	unpaired t-test
Mouse PD-L1	DMSO vs 100nM	0.6581	unpaired t-test
	DMSO vs 1µM	0.3118	unpaired t-test

Fig EV3D	P-value	Test
PD-L1/DMSO<br>vs CON/DMSO	<0.0001	unpaired t-test
PD-L1/DMSO<br>vs PD-L1/100nM MB	0.0001	unpaired t-test
PD-L1/DMSO<br>vs PD-L1/1µM MB	<0.0001	unpaired t-test

Fig EV3E	P-value	Test
PD-L1/DMSO<br>vs CON/DMSO	0.0011	unpaired t-test
PD-L1/DMSO<br>vs PD-L1/100nM MB	0.0082	unpaired t-test
PD-L1/DMSO<br>vs PD-L1/1µM MB	0.0003	unpaired t-test

Fig EV3G	P-value	Test
PVD/DMSO<br>vs CON/DMSO	0.0003	unpaired t-test
PVD/DMSO<br>vs PVD/100nM MB	0.0047	unpaired t-test
PVD /DMSO<br>vs PVD /1µM MB	0.0006	unpaired t-test

Fig EV3H	P-value	Test
PVD/DMSO<br>vs CON/DMSO	<0.0001	unpaired t-test
PVD/DMSO<br>vs PVD/100nM MB	0.0006	unpaired t-test
PVD /DMSO<br>vs PVD /1µM MB	<0.0001	unpaired t-test

Fig EV3L	P-value	Test
PD-1-Cluc-Y248F<br>vs PD-1-Cluc	0.0002	unpaired t-test

Fig EV3M	P-value	Test
DMSO vs 100nM	<0.0001	unpaired t-test
DMSO vs 500nM	<0.0001	unpaired t-test
DMSO vs 1µM	<0.0001	unpaired t-test
DMSO vs 2µM	<0.0001	unpaired t-test

Fig EV3R		P-value	Test

JP-luc sgSHP2	Raji PD-L1 vs Raji	0.5529	unpaired t-test
	Raji PD-L1 vs Raji PD-L1+aPD1	0.7102	unpaired t-test
	Raji PD-L1 vs Raji PD-L1+MB	0.2098	unpaired t-test
JP-luc SHP099	Raji PD-L1 vs Raji	0.1446	unpaired t-test
	Raji PD-L1 vs Raji PD-L1+aPD1	0.6664	unpaired t-test
	Raji PD-L1 vs Raji PD-L1+MB	0.1929	unpaired t-test

Fig EV3T		P-value	Test
sgSHP2	PD-L1/DMSO vs CON/DMSO	0.0227	unpaired t-test
	PD-L1/DMSO vs PD-L1/aPD1	0.0392	unpaired t-test
	PD-L1/DMSO vs PD-L1/MB	0.0173	unpaired t-test
SHP099	PD-L1/DMSO vs CON/DMSO	0.4775	unpaired t-test
	PD-L1/DMSO vs PD-L1/aPD1	0.9235	unpaired t-test
	PD-L1/DMSO vs PD-L1/MB	0.6190	unpaired t-test

Fig EV3U		P-value	Test
sgSHP2	PD-L1/DMSO vs CON/DMSO	0.5505	unpaired t-test
	PD-L1/DMSO vs PD-L1/aPD1	0.0542	unpaired t-test
	PD-L1/DMSO vs PD-L1/MB	0.0074	unpaired t-test
SHP099	PD-L1/DMSO vs CON/DMSO	0.0165	unpaired t-test
	PD-L1/DMSO vs PD-L1/aPD1	0.0008	unpaired t-test
	PD-L1/DMSO vs PD-L1/MB	0.0057	unpaired t-test

Fig EV3V		P-value	Test
sgSHP2	EG7 PD-L1/DMSO vs EG7/DMSO	0.2444	unpaired t-test
	EG7 PD-L1/DMSO	0.1917	unpaired t-test

	vs EG7 PD-L1/aPD1		
	EG7 PD-L1/DMSO vs EG7 PD-L1/MB	0.5053	unpaired t-test
SHP099	EG7 PD-L1/DMSO vs EG7/DMSO	0.2155	unpaired t-test
	EG7 PD-L1/DMSO vs EG7 PD-L1/aPD1	0.4226	unpaired t-test
	EG7 PD-L1/DMSO vs EG7 PD-L1/MB	0.0356	unpaired t-test

Fig EV3Y	P-value	Test
DMSO vs 10nM	0.6296	unpaired t-test
DMSO vs 100nM	0.2871	unpaired t-test
DMSO vs 500nM	0.9479	unpaired t-test
DMSO vs 1μM	0.9715	unpaired t-test

Fig EV3Z	P-value	Test
DMSO vs 10nM	0.3721	unpaired t-test
DMSO vs 100nM	0.5735	unpaired t-test
DMSO vs 500nM	0.6380	unpaired t-test
DMSO vs 1μM	0.0871	unpaired t-test

Fig EV4A	Days after tumor injection	P-value	Test
Vehicle vs 10mg/kgMB	11	0.0082	unpaired t-test
Vehicle vs 20mg/kgMB	11	0.0073	unpaired t-test
Vehicle vs 30mg/kgMB	11	0.0041	unpaired t-test

Fig EV4C	Days after tumor injection	P-value	Test
Vehicle vs aPD1	11	0.0122	unpaired t-test
	13	0.0439	unpaired t-test
Vehicle vs MB	11	0.0072	unpaired t-test
	13	0.0456	unpaired t-test

Fig EV4E	P-value	Test
Vehicle vs aPD1	0.0089	unpaired t-test
Vehicle vs MB	0.0033	unpaired t-test

Fig EV4F	Days after tumor injection	P-value	Test
Vehicle vs 10mg/kg MB	13	0.04	unpaired t-test
Vehicle vs 20mg/kg MB	13	0.029	unpaired t-test
Vehicle vs 30mg/kg MB	13	0.024	unpaired t-test

Fig EV4I	Days after tumor injection	P-value	Test
WT CTL Vehicle vs WT CTL aPD1	9	0.0040	unpaired t-test
	11	0.0031	unpaired t-test
WT CTL Vehicle vs WT CTL MB	9	0.0043	unpaired t-test
	11	0.0035	unpaired t-test
PD-1 KO CTL Vehicle vs PD-1 KO CTL aPD1	9	0.8711	unpaired t-test
	11	0.2096	unpaired t-test
PD-1 KO CTL Vehicle vs PD-1 KO CTL MB	9	0.1734	unpaired t-test
	11	0.2486	unpaired t-test

Fig EV4K		P-value	Test
WT CTL	Vehicle vs aPD1	0.0010	unpaired t-test
	Vehicle vs MB	0.0012	unpaired t-test
PD-1 KO CTL	Vehicle vs aPD1	0.2058	unpaired t-test
	Vehicle vs MB	0.0570	unpaired t-test

Fig EV4L		P-value	Test
DOX+/+	0d vs 5d	0.0035	unpaired t-test
DOX+/-	0d vs 5d	0.0004	unpaired t-test

Fig EV4M	P-value	Test
DOX+/+ vs DOX+/-	0.0238	unpaired t-test

Fig EV4O		P-value	Test
Vehicle	PreRx vs PostRx	0.0178	unpaired t-test
aPD1	PreRx vs PostRx	0.1435	unpaired t-test

Fig EV4P	P-value	Test
Vehicle vs aPD1	0.2771	unpaired t-test

Fig EV4S		P-value	Test
IL-2	Vehicle vs aPD1	<0.0001	unpaired t-test
Perforin	Vehicle vs aPD1	0.0005	unpaired t-test
GZMB	Vehicle vs aPD1	0.0002	unpaired t-test

Fig EV5B		P-value	Test
24h	PD-L1/DMSO vs CON/DMSO	0.0001	unpaired t-test
	PD-L1/DMSO vs PD-L1/Pem	0.0099	unpaired t-test
	PD-L1/DMSO vs PD-L1/Niv	0.0289	unpaired t-test
	PD-L1/DMSO vs PD-L1/MB	0.0043	unpaired t-test
48h	PD-L1/DMSO vs CON/DMSO	0.0025	unpaired t-test
	PD-L1/DMSO vs PD-L1/Pem	0.0035	unpaired t-test
	PD-L1/DMSO vs PD-L1/Niv	0.0154	unpaired t-test
	PD-L1/DMSO vs PD-L1/MB	0.0068	unpaired t-test

Fig EV5C	P-value	Test
PD-L1/DMSO vs CON/DMSO	0.0045	unpaired t-test
PD-L1/DMSO vs PD-L1/Pem	0.0009	unpaired t-test
PD-L1/DMSO vs PD-L1/Niv	0.0211	unpaired t-test
PD-L1/DMSO vs PD-L1/100nM MB	0.0023	unpaired t-test
PD-L1/DMSO vs PD-L1/1μM MB	0.0006	unpaired t-test

Fig EV5D	P-value	Test
PD-L1/DMSO vs CON/DMSO	0.0010	unpaired t-test
PD-L1/DMSO vs PD-L1/Pem	0.0005	unpaired t-test
PD-L1/DMSO vs PD-L1/Niv	0.0333	unpaired t-test
PD-L1/DMSO vs PD-L1/100nM MB	0.0044	unpaired t-test
PD-L1/DMSO vs PD-L1/1µM MB	0.0005	unpaired t-test

Fig EV5E	P-value	Test
PD-L1/DMSO vs CON/DMSO	0.0009	unpaired t-test
PD-L1/DMSO vs PD-L1/Pem	0.0021	unpaired t-test
PD-L1/DMSO vs PD-L1/Niv	0.0009	unpaired t-test
PD-L1/DMSO vs PD-L1/100nM MB	0.0032	unpaired t-test
PD-L1/DMSO vs PD-L1/1µM MB	0.0005	unpaired t-test

Fig EV5F	P-value	Test
PD-L1/DMSO vs CON/DMSO	0.0076	unpaired t-test
PD-L1/DMSO vs PD-L1/Pem	0.0043	unpaired t-test
PD-L1/DMSO vs PD-L1/Niv	0.0046	unpaired t-test
PD-L1/DMSO vs PD-L1/100nM MB	0.0035	unpaired t-test
PD-L1/DMSO vs PD-L1/1µM MB	0.0002	unpaired t-test

*P < 0.05; **P < 0.01; ***P < 0.001; ****P < 0.0001

Appendix Table S2: Antibody dilution list

Antibody	Company	Catalog Number	Dilution
CD8-PE anti-mouse	eBioscience	12-0081-81	1/100
IFNγ-PE-Cy7 anti-mouse	eBioscience	25-7311-82	1/100
Perforin–APC anti-mouse	eBioscience	17-9392-80	1/100
CD8-FITC anti-mouse	eBioscience	11-0081-82	1/100
IL-2-PE PE anti-mouse	eBioscience	12-7021-82	1/100
CD25-PE anti-mouse	eBioscience	12-0691-81	1/100
CD69-PE anti- mouse	eBioscience	12-0251-81	1/100
PD-1-FITC anti-mouse	eBioscience	11-9985-85	1/100
PD-L1-PE anti-mouse	eBioscience	12-5982-81	1/100
CD8-BUV737 anti-mouse	BD Horizon	564297	1/100
CD8-PE anti-human	eBioscience	MHCD0804	1/100
GZMB anti-mouse/human	Biolegend	515405	1/100
IL-2-PE anti-human	Biolegend	500306	1/100
IFNγ-PE anti-human	Biolegend	502508	1/100
Perforin-PE anti-human	Biolegend	353303	1/100
CD25-PE anti-human	Biolegend	356103	1/100
CD69-PE anti-human	Biolegend	310905	1/100
CD8-FITC anti-human	Biolegend	300906	1/100
CD45.1-FITC anti-mouse	Biolegend	110705	1/100
CD45.2-APC anti-mouse	Biolegend	109813	1/100
PD-1-FITC anti-human	Biolegend	329904	1/100
PD-1-PE anti-human	Biolegend	329905	1/100
PD-L1-PE anti-human	Biolegend	329706	1/100
His Tag-PE	Biolegend	362603	1/100
His-HRP	Biolegend	652504	1/5000
FC-HRP	Sino biological	SSA001	1/5000
CD28 anti-human	ebiocience	16-0289	2ug/mL
CD3 anti-human	ebiocience	16-0037	2ug/mL
CD28 anti-mouse	ebiocience	16-0281	2ug/mL
CD3 anti-mouse	ebiocience	16-0031	2ug/mL
Phospho-Zap-70	Cell Signaling Technology	2717	1/1000
ZAP70	Cell Signaling Technology	3165	1/1000
Phospho-PKCθ	Cell Signaling Technology	9377	1/1000
PKCθ	Cell Signaling Technology	13643	1/1000
Phospho-PLCγ1	Cell Signaling Technology	14008	1/1000
PLCγ1	Cell Signaling Technology	5690	1/1000
Phospho-AKT	Cell Signaling Technology	4060S	1/1000
AKT	Cell Signaling Technology	9272S	1/1000
PD-1 human	Cell Signaling Technology	86163	1/1000
PD-1 mouse	Cell Signaling Technology	84651	1/1000

PD-L1 mouse	Cell Signaling Technology	13684	1/1000
SHP2	Cell Signaling Technology	3397	1/1000
Phospho-Tyrosine Mouse mAb (P-Tyr-102)	Cell Signaling Technology	9416	1/1000
CD28	Cell Signaling Technology	38774	1/1000
CD8α Mouse Specific	Cell Signaling Technology	98941	1/50
ki-67	Abcam	ab15580	1/500
β-Actin	sigma	A5316	1/5000
EGFR	Abcam	E234	1/1000
HA	Santa Cruz	C1091	1/1000
Flag	sigma	F1804	1/1000
Mouse PD-1 antibody	BioXcell	BP0033-2	10mg/kg
Mouse CD8 antibody	BioXcell	BE0061	10mg/kg
Anti-mouse IgG	sigma	A0168	1/2000
Anti-Rabbit IgG	sigma	A6154	1/2000
