# Supplementary material for: Blocking interaction between SHP2 and PD‐1 denotes a novel opportunity for developing PD‐1 inhibitors
Source: EMBO Mol Med. 2020 May 11;12(6):e11571. doi: 10.15252/emmm.201911571 (PMC7278553; doi:10.15252/emmm.201911571)
Supplement: Supplementary file 2 — Expanded View Figures PDF [file EMMM-12-e11571-s002.pdf]

Expanded View Figures

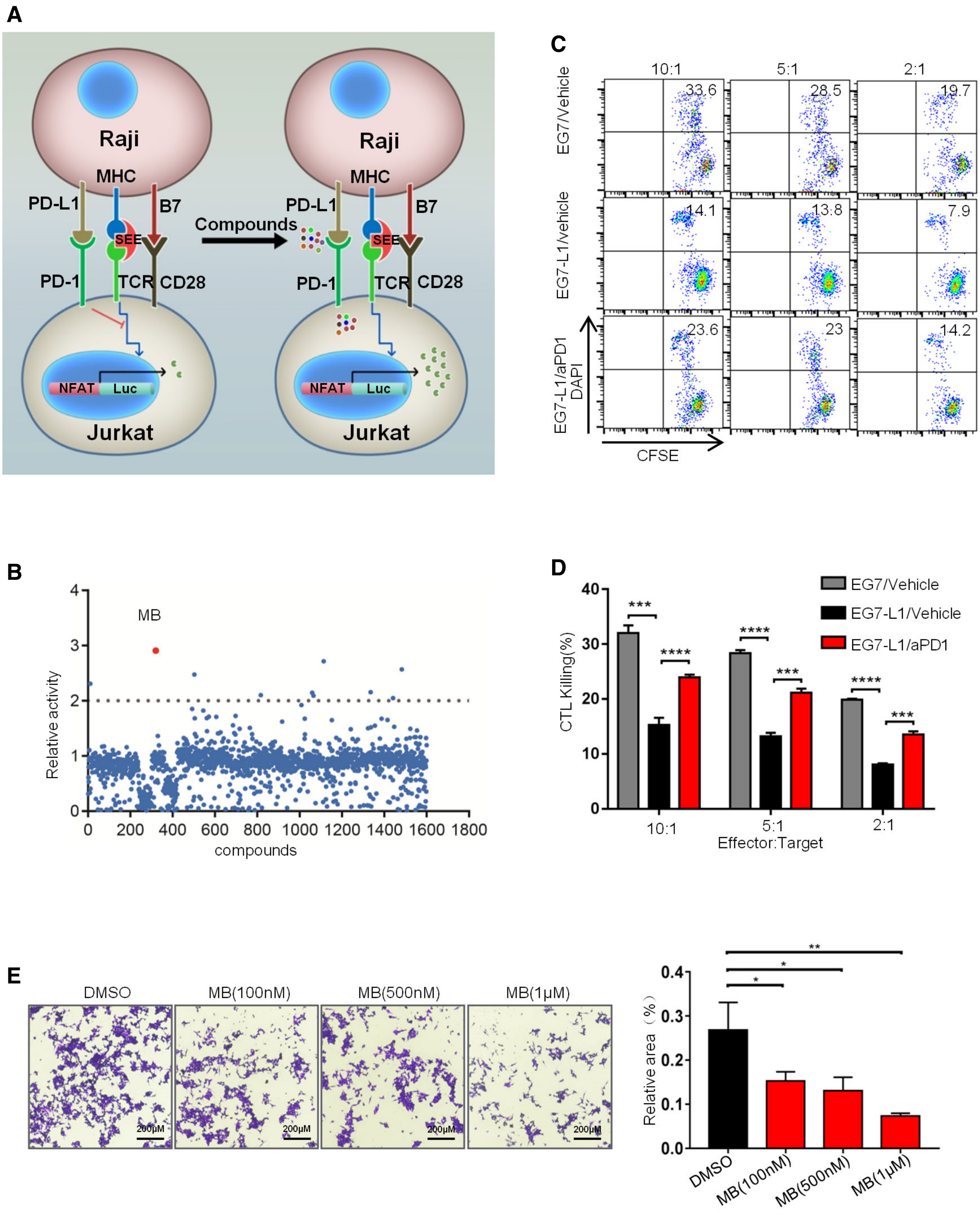

Figure EV1.

 © 2020 The Authors

Figure EV1. MB enhances cytotoxicity of activated CTL against PD-L1 expressing target cells.

A Schematic diagram for compounds screening on Jurkat and Raji stable lines.
B Scatterplot of relative luciferase activity of JP-luc stimulated with SEE-loaded Raji-L1 in the presence of 1 μM chemicals. Y axis denotes the ratio of luciferase of chemical treated well against that of DMSO treated well. JP-luc: Jurkat cell harboring NFAT-luciferase transgene and overexpressing PD-1; Raji-L1: Raji overexpressing PD-L1.
C FACS analysis of the cytotoxic efficiency of OT-I CTLs against EG7-L1 in the presence of MB. aPD1 antibody served as positive control. Splenocytes from OT-I mice in culture were stimulated with 10 nM of SINFEEKL peptide for 3 days to generate mature CTLs. CTLs were incubated with CFSE-labeled EG7-L1 cells in the presence of MB at indicated concentrations. Cytotoxicity was determined by flow cytometry. Data are representative of three independent experiments (effector-to-target ratio = 10:1, 5:1, 2:1, unpaired *t*-test, killing time: 5 h). EG7-L1: EG7 overexpressing PD-L1.
D Statistical results of (Fig EV1C).
E MB enhanced cytotoxicity of OT-I CTLs against IFNγ-treated B16-F10-OVA. Cytotoxicity was determined by the relative area unoccupied by crystal violet stained cells examined under microphotograph.

Data information: Data are representative of three independent experiments and were analyzed by unpaired *t*-test. Error bars denote SEM. $*P < 0.05$; $**P < 0.01$; $***P < 0.001$; $****P < 0.0001$.

© 2020 The Authors

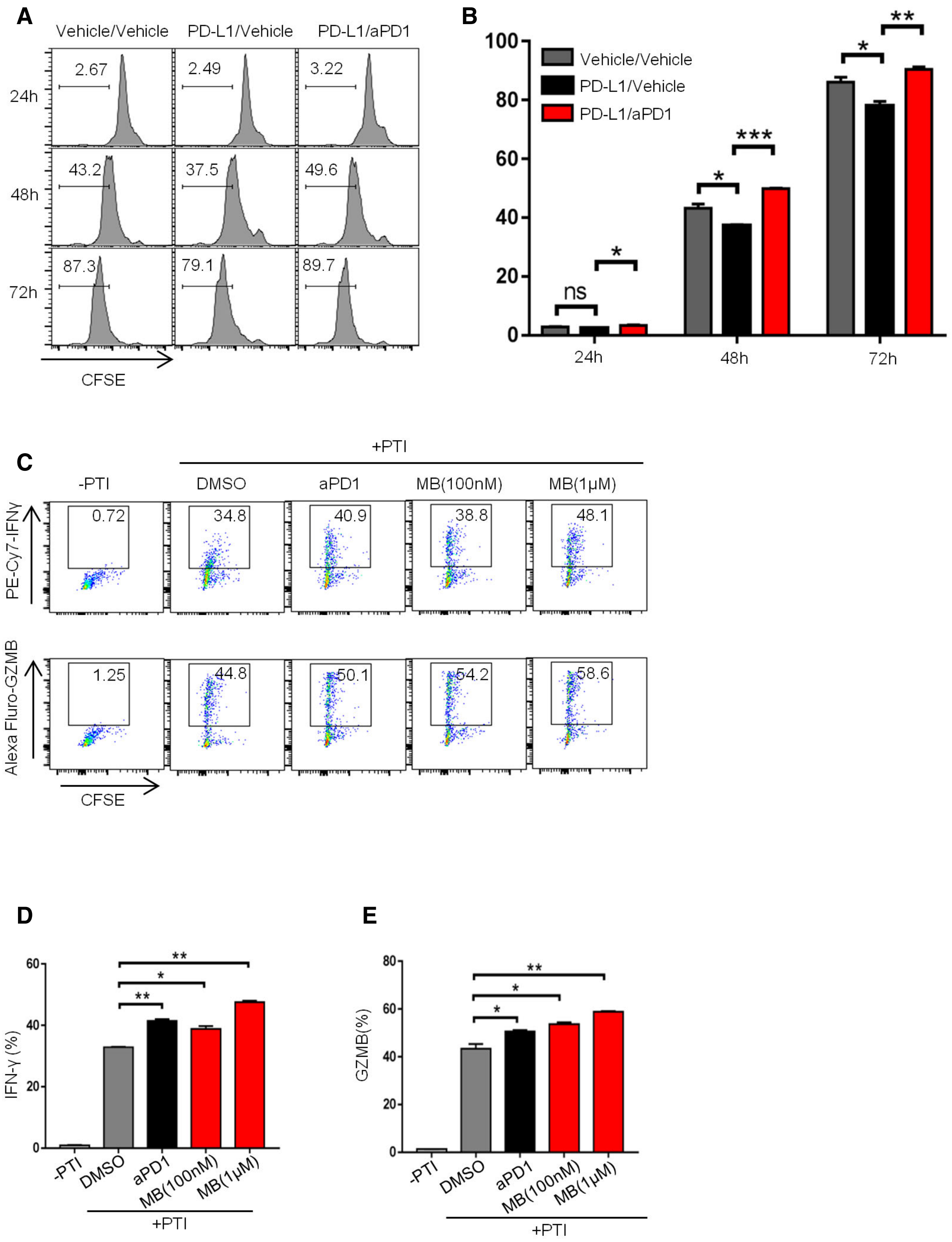

Figure EV2.

© 2020 The Authors

Figure EV2. MB enhanced activation and effector function of CTL.

A Effect of PD-1 antibody on the proliferation of OT-I CTLs. Splenocytes from OT-I mice were labeled with CFSE and seeded in a 96-well plate. Media were supplemented with 10 nM of SINFEEKL peptide, 10 ng/ml of human IL-2 and 10 μg/ml of mouse PD-L1 protein in the presence of 10 μg/ml of PD-1 antibody. Cell proliferation was measured by FACS.
B Bar graph of (Fig EV2A).
C MB enhancing production of cytokine and cytolytic granule by OT-1 CTLs. CTLs were co-incubated with CFSE-labeled EG7-L1 cells in the presence of protein transport inhibitor (PTI) and MB at indicated concentrations. Expression of cytokine and cytolytic granule was determined by flow cytometry. aPD-1 antibody served as positive control. EG7-L1: EG7 overexpressing PD-L1.
D Bar graph of IFNγ production of OT-1 CTLs in (Fig EV2C).
E Bar graph of GZMB production of OT-1 CTLs in (Fig EV2C).

Data information: Data are representative of three independent experiments and were analyzed by unpaired *t*-test. Error bars denote SEM. *$P < 0.05$; **$P < 0.01$; ***$P < 0.001$.

© 2020 The Authors

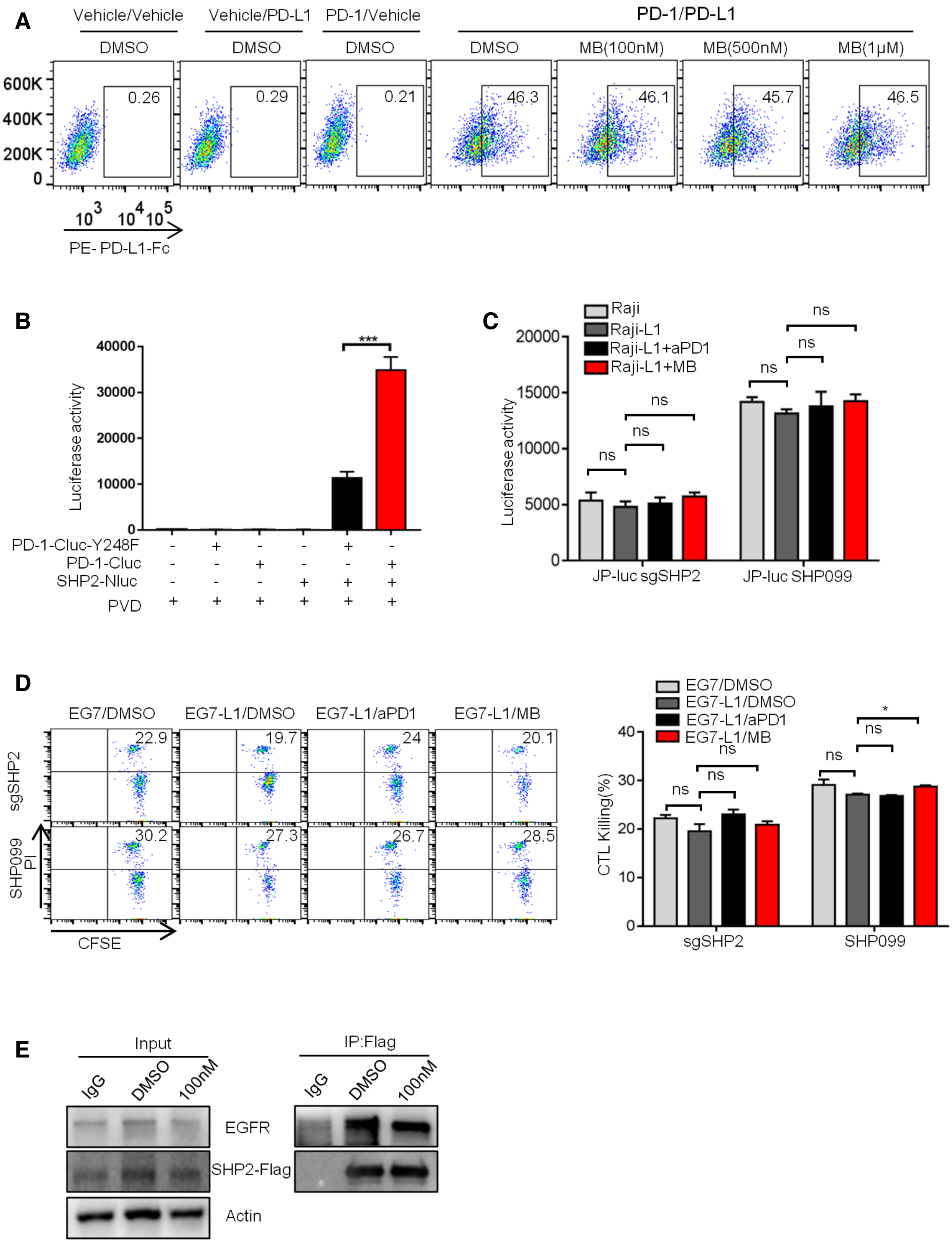

Figure EV3.

 © 2020 The Authors

Figure EV3. MB suppress PD-1 signaling through blocking SHP2 recruitment by PD-1.

A Impact of MB on the interaction between PD-1 and PD-L1. Jurkat-PD-1 cells (parental Jurkat cells served as negative control) were incubated with human PD-L1-Fc fusion protein in the presence of MB at indicated concentration at 4 for 30 min. Cells were stained for PE-conjugated anti-hIgG-Fc antibody. Binding of PD-L1 on cells was determined by flow cytometry.

B Luciferase complementation analysis showing the effect of PD-1 Y248F mutation on the interaction between PD-1 and SHP2.

C Impact of MB on luciferase activity of various engineered Jurkat T cells. JP-luc-sgSHP2: JP-luc cells treated with lentivirus expressing sgSHP2/CAS9 simultaneously. SHP099: JP-luc cells treated with 10 μM SHP099. JP-luc: Jurkat cell harboring NFAT-luciferase transgene and overexpressing PD-1.

D Impact of MB on cytotoxicity of sgSHP2 or OT-1 in the presence of 10 μM SHP099 against EG7 or EG7-L1. EG7-L1: EG7 overexpressing PD-L1.

E Co-IP analysis of impact of MB on interaction between SHP2 and EGFR.

Data information: Data are representative of three independent experiments and were analyzed by unpaired *t*-test. Error bars denote SEM. *$P < 0.05$; ***$P < 0.001$.

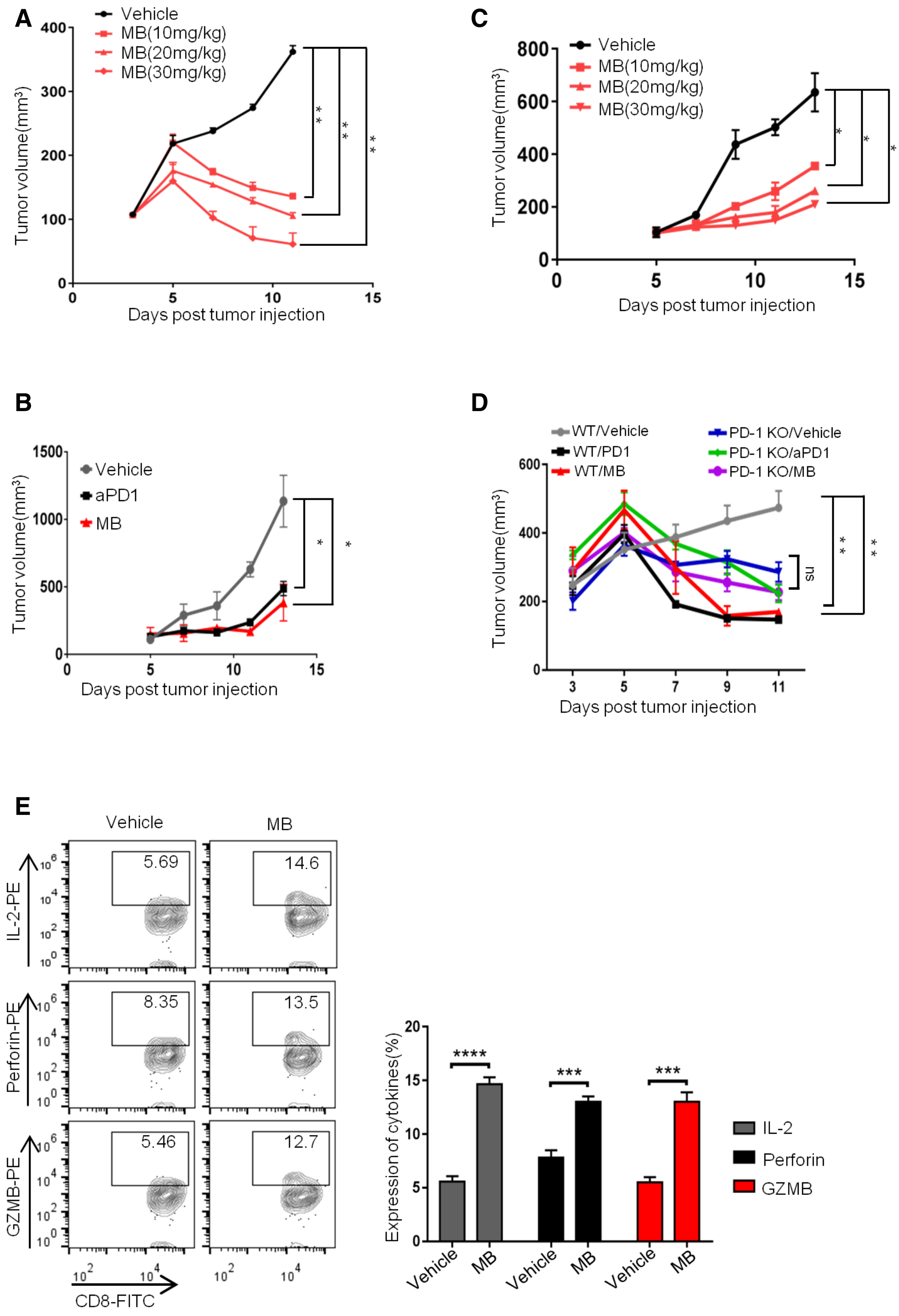

Figure EV4.

© 2020 The Authors

Figure EV4. MB shrinks tumor *in vivo* through enhancing cytotoxic function of CTL.

A Effect of different concentration of MB on EG7-L1 xenograft in C57BL/6J mice (n = 5). EG7-L1: EG7 overexpressing PD-L1.
B EG7-L1 xenograft growth curve in RAG1$^{-/-}$ mice (n = 2).
C Effect of different concentration of MB on xenograft in RAG1$^{-/-}$ mice (n = 2).
D Tumor growth curves of subcutaneous tumor allograft. EG7-L1 cell (2 × 10^6 cells) was subcutaneously injected into the right flank of C57BL/6J mice (n = 4 per group). The mice were then injected with WT or PD-1KO CTLs (i.v.) on days 3 and 6, respectively, and treated with vehicle, aPD1 (i.*p*. 10 mg/kg, every other day), or MB (i.g. 20 mg/ kg/day). Tumor volume is shown as mean ± SEM.
E Expression of IL-2, granzyme B, and perforin by CD8$^+$ T Cells was analyzed in tumors of EC mice after treatment with MB by FACS.

Data information: Data are representative of three independent experiments and were analyzed by unpaired *t*-test. Error bars denote SEM. *$P < 0.05$; **$P < 0.01$; ***$P < 0.001$; ****$P < 0.0001$.

© 2020 The Authors

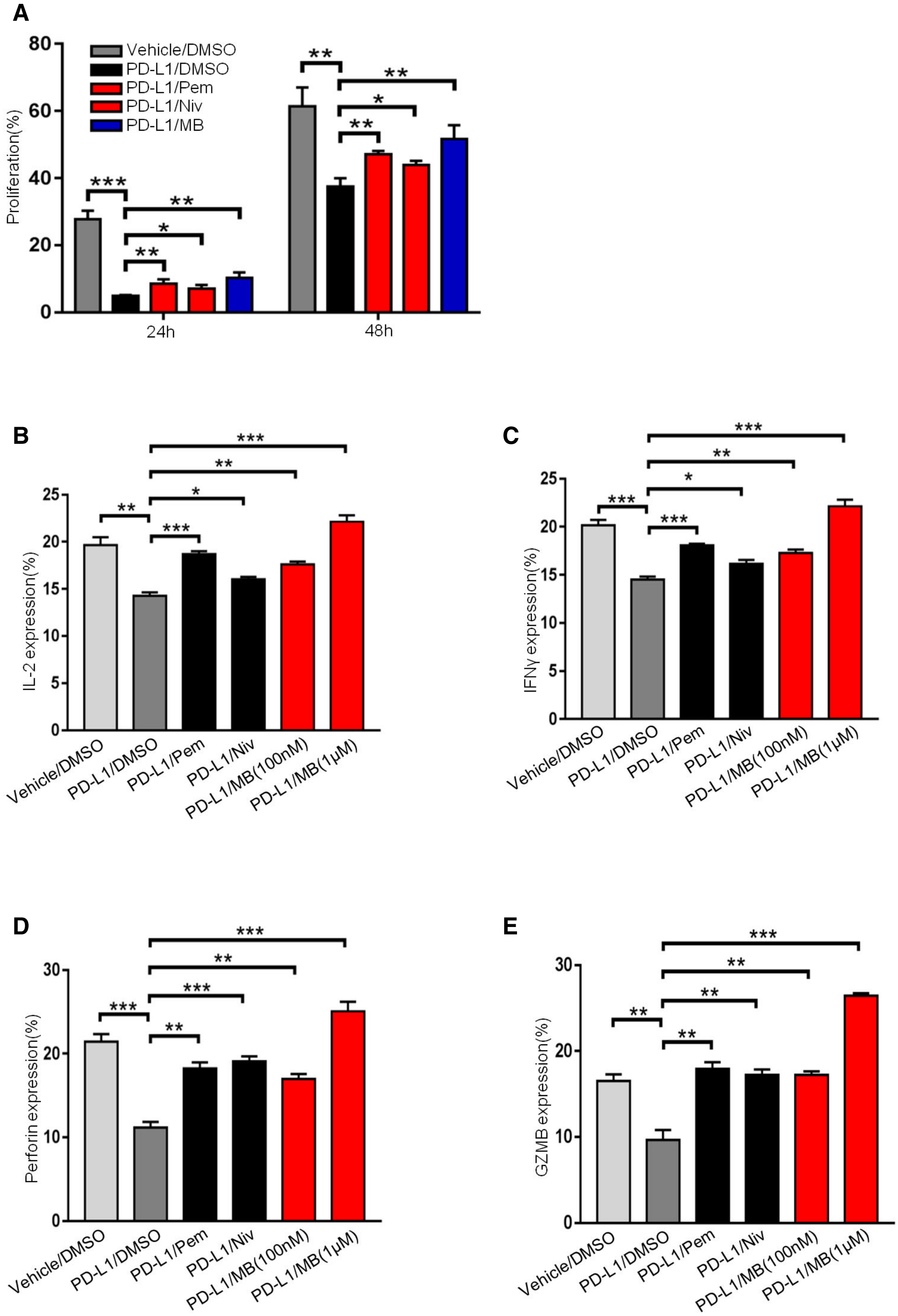

Figure EV5.

 © 2020 The Authors

Figure EV5. MB is effective to activate human CD8$^+$ T cells.

A Bar graph of proliferation of human CD8 T cells.
B Bar graph of IL-2 production of human CD8$^+$ T cells.
C Bar graph of IFNγ production of human CD8$^+$ T cells.
D Bar graph of perforin production of human CD8$^+$ T cells.
E Bar graph of GZMB production of human CD8$^+$ T cells.

Data information: Data are representative of three independent experiments and were analyzed by unpaired *t*-test. Error bars denote SEM. *$P < 0.05$; **$P < 0.01$; ***$P < 0.001$.

© 2020 The Authors
