# Supplementary material for: Blocking interaction between SHP2 and PD‐1 denotes a novel opportunity for developing PD‐1 inhibitors
Source: EMBO Mol Med. 2020 May 11;12(6):e11571. doi: 10.15252/emmm.201911571 (PMC7278553; doi:10.15252/emmm.201911571)

Figure S1C

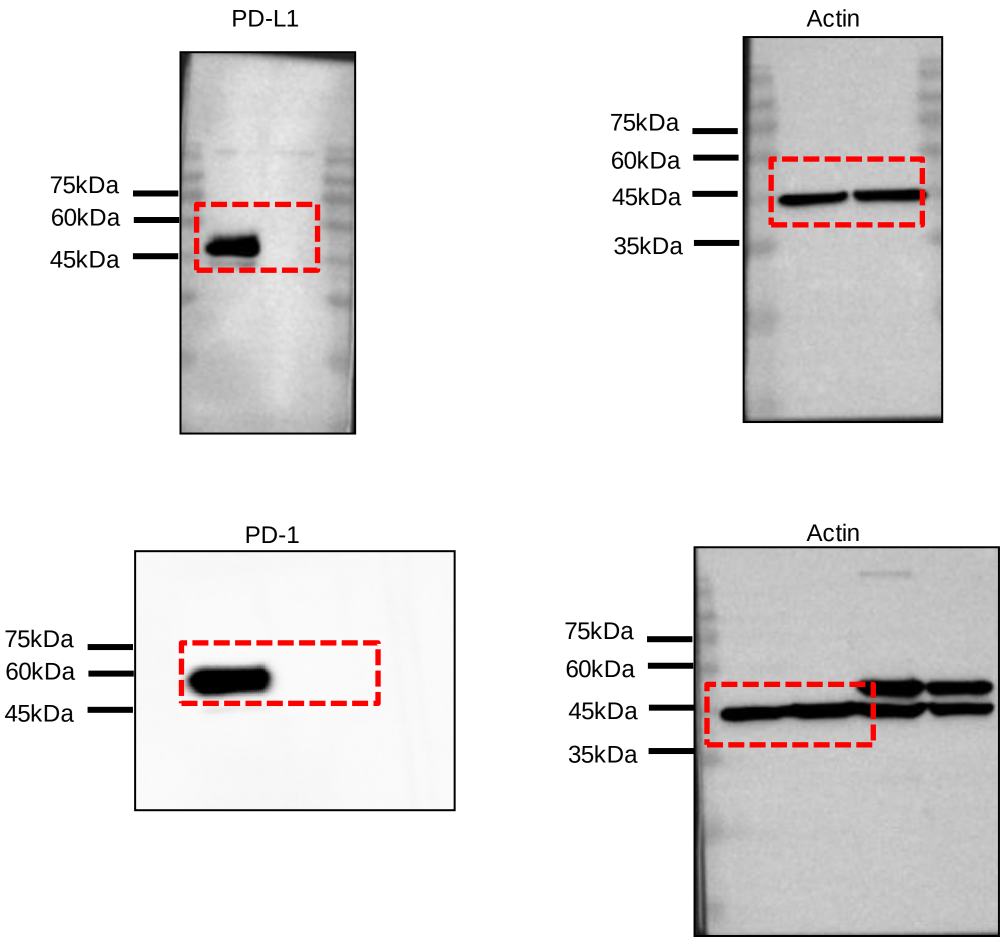
PD-L1
75kDa
60kDa
45kDa
Actin
75kDa
60kDa
45kDa
35kDa
PD-1
75kDa
60kDa
45kDa
Actin
75kDa
60kDa
45kDa
35kDa

Figure S1F

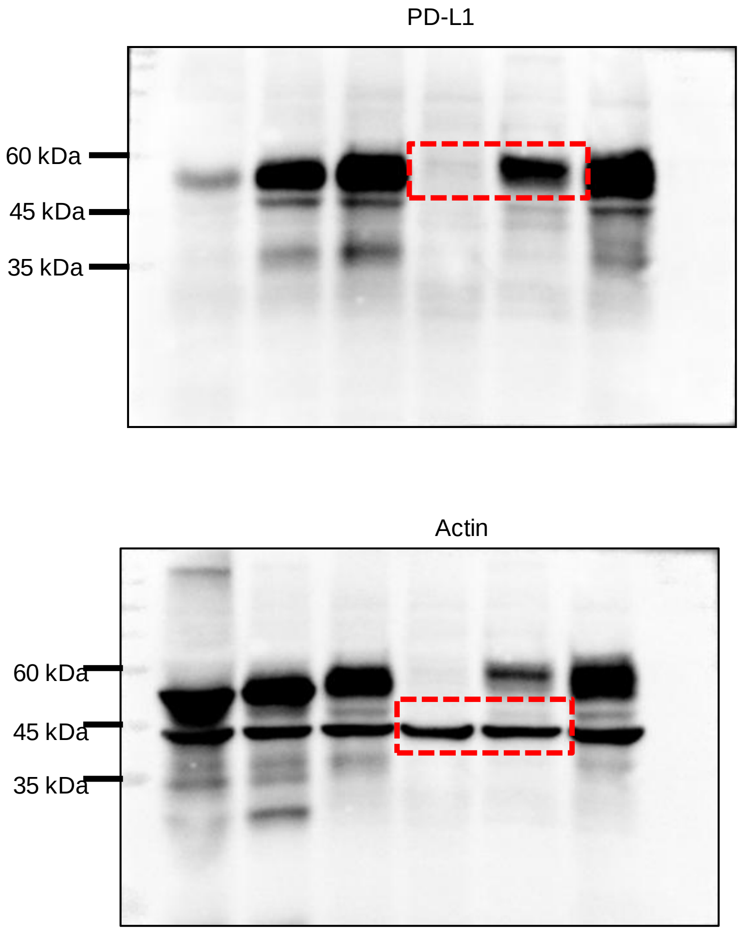

Figure S1P

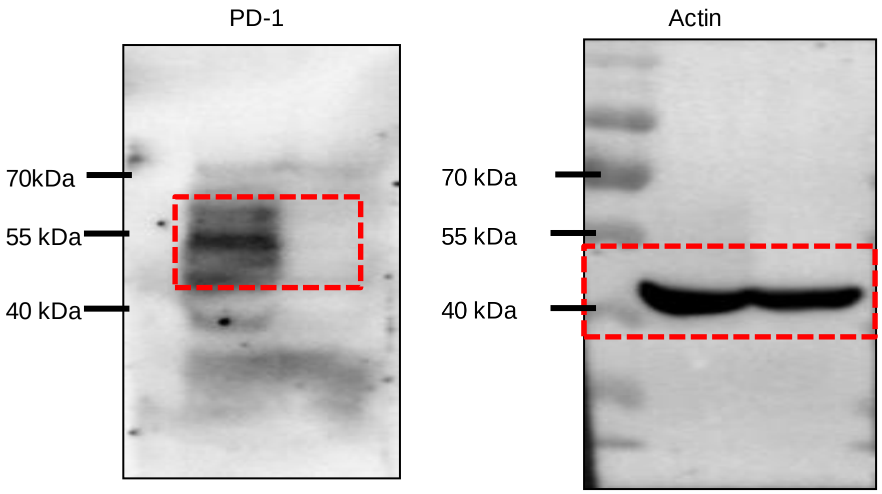

Figure S2E

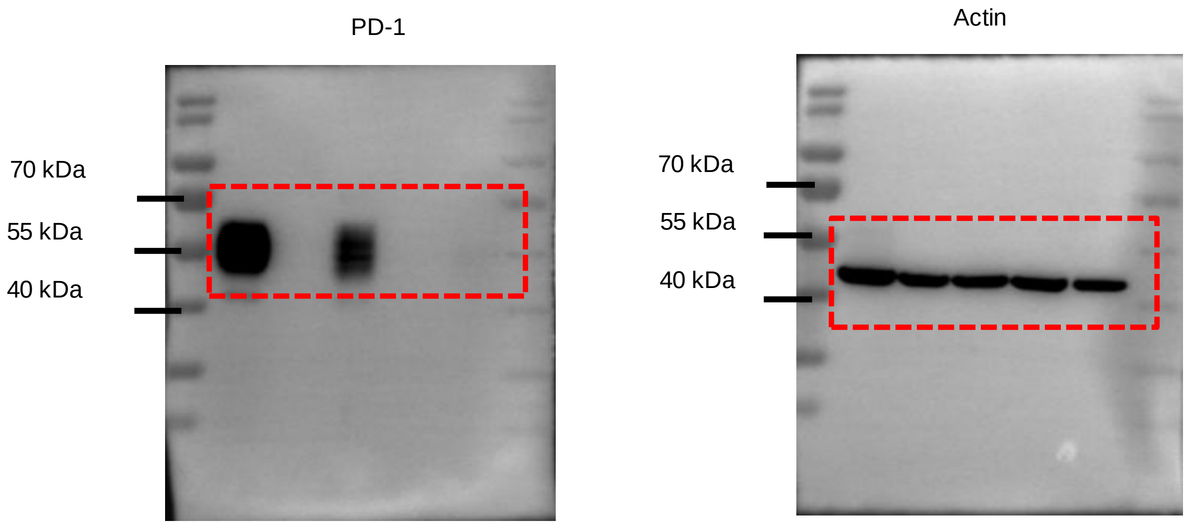

Figure S3I, S3J

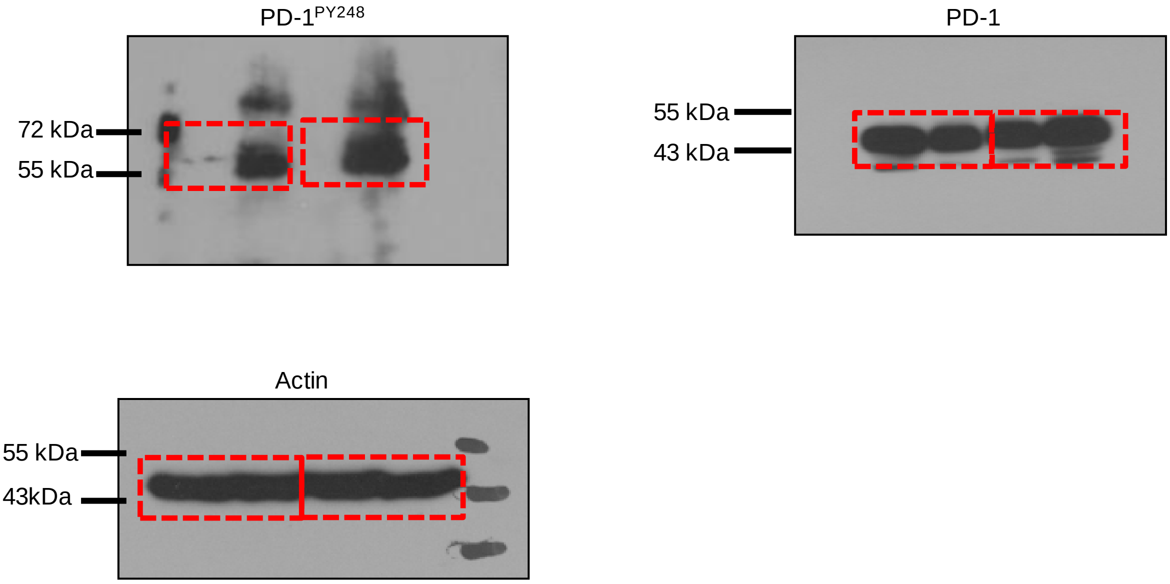

Figure S3K

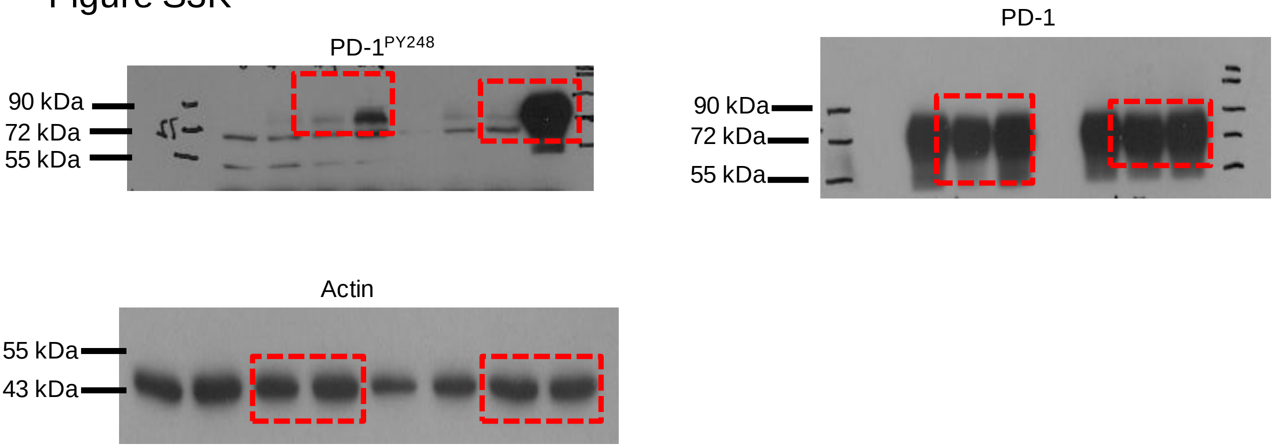

Figure S3Q

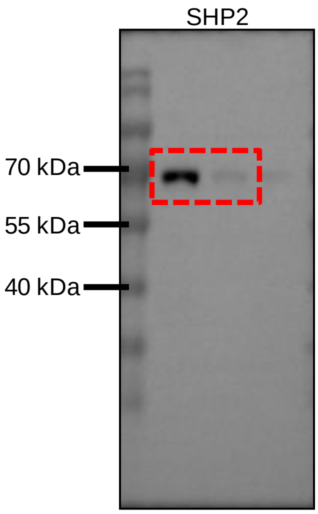

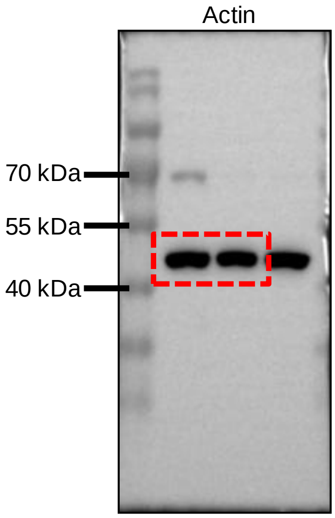

Figure S3S

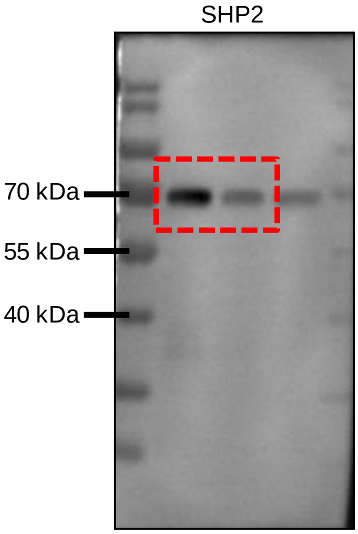

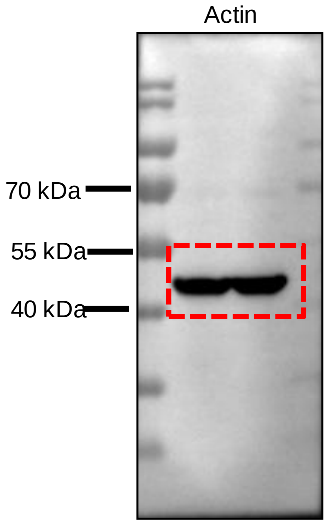

Figure S3W

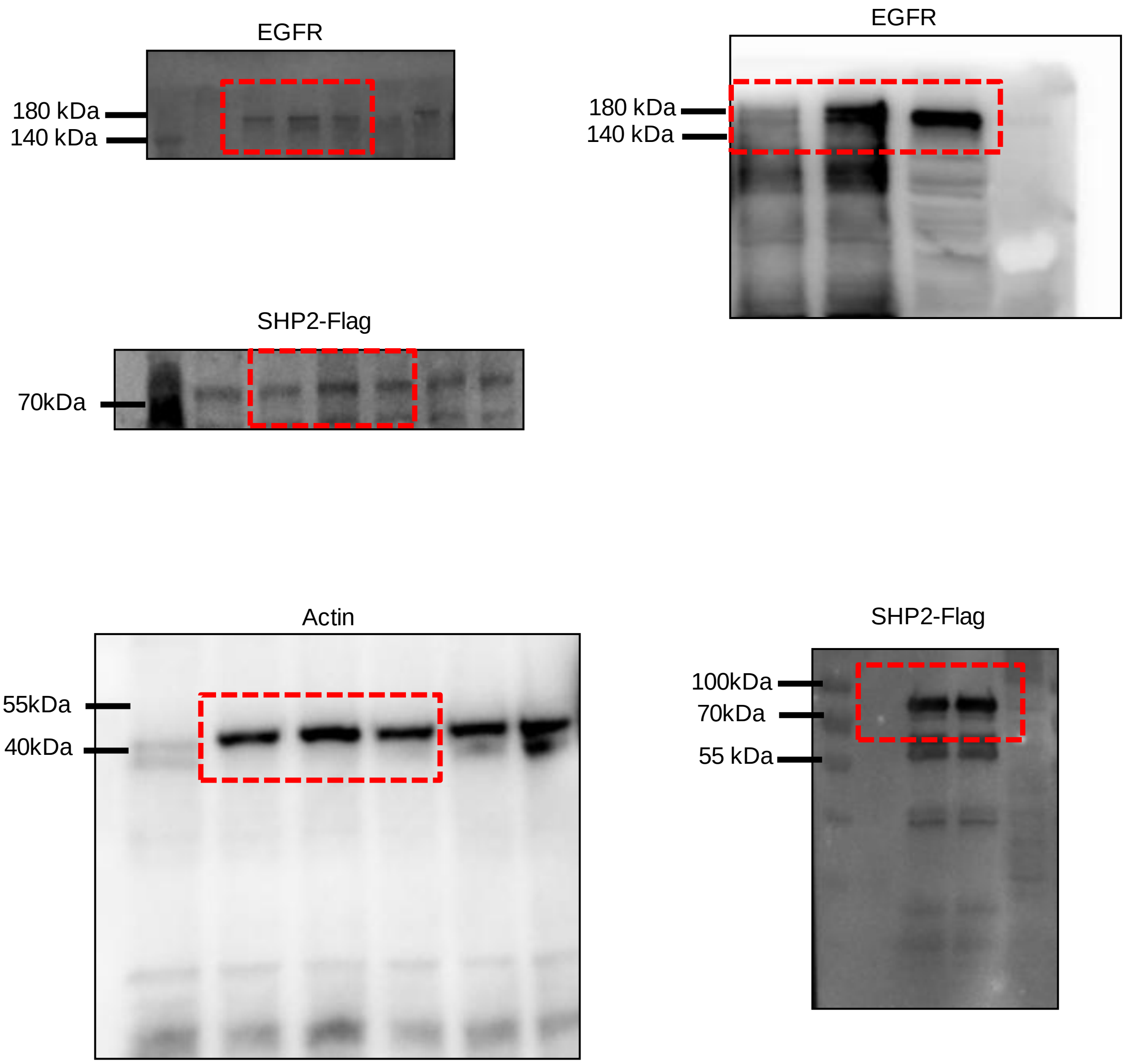
EGFR
180 kDa
140 kDa
EGFR
180 kDa
140 kDa
SHP2-Flag
70kDa
Actin
55kDa
40kDa
SHP2-Flag
100kDa
70kDa
55 kDa

Figure S3X

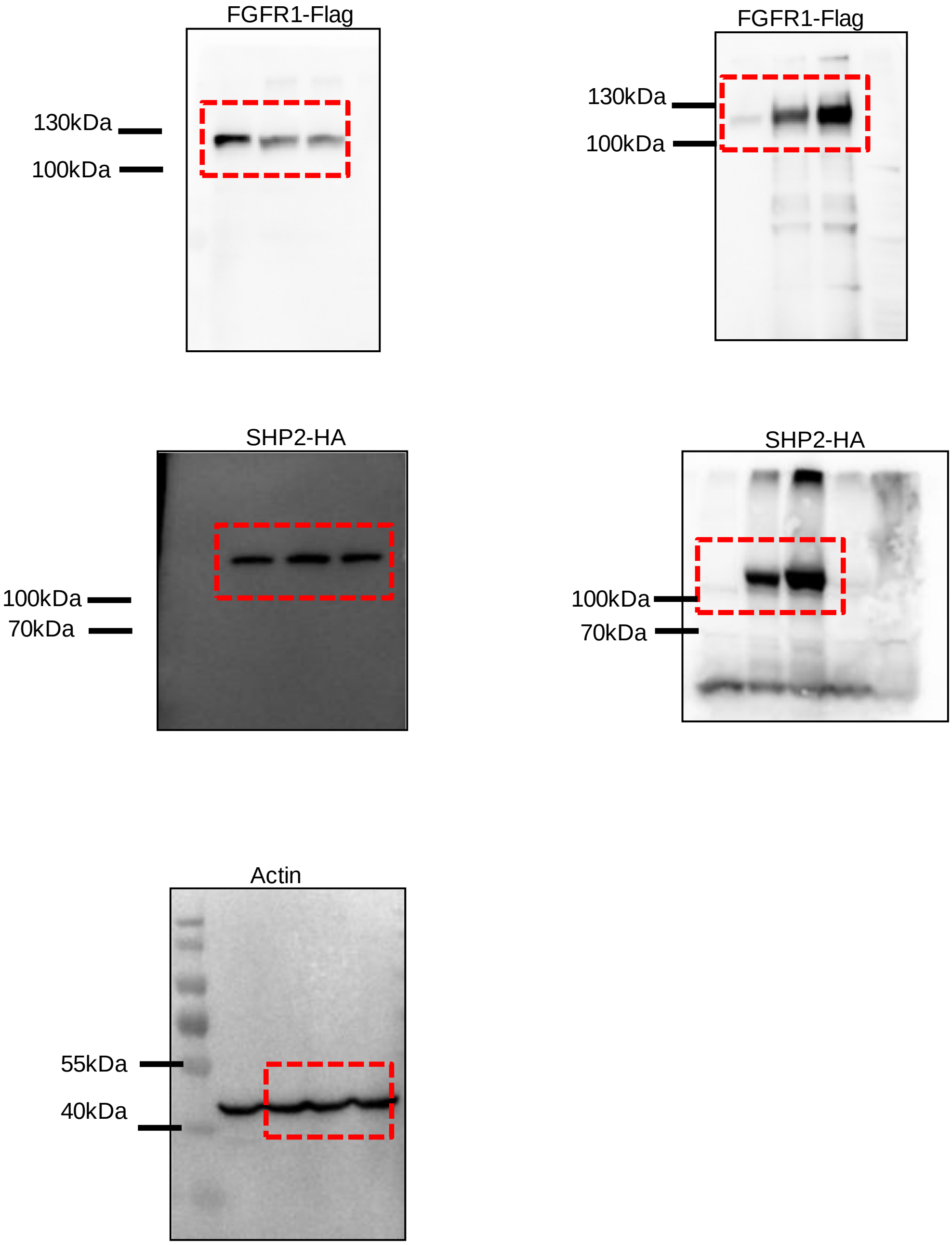
FGFR1-Flag
130kDa
100kDa
FGFR1-Flag
130kDa
100kDa
SHP2-HA
100kDa
70kDa
SHP2-HA
100kDa
70kDa
Actin
55kDa
40kDa

Figure S4N

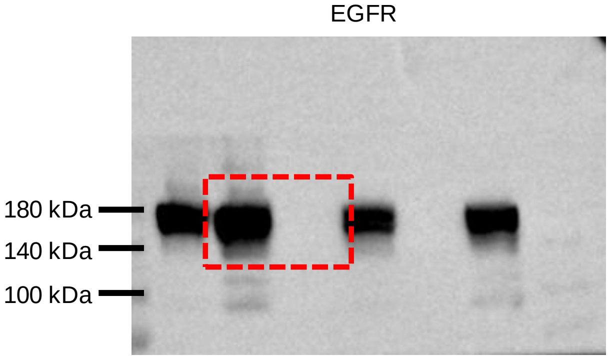

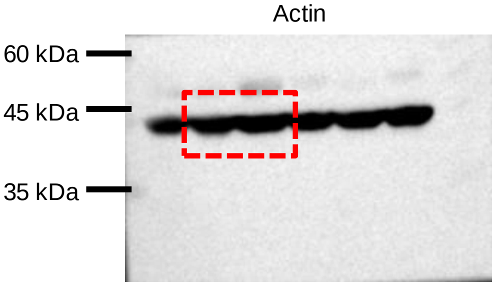

Supplement: Supplementary file 3 — Source Data for Appendix [file EMMM-12-e11571-s004.zip › Source_Data_for_Appendix_Figures.pdf]
