# Supplementary figures and images for: Blocking interaction between SHP2 and PD‐1 denotes a novel opportunity for developing PD‐1 inhibitors
Source: EMBO Mol Med. 2020 May 11;12(6):e11571. doi: 10.15252/emmm.201911571 (PMC7278553; doi:10.15252/emmm.201911571)

Figure 3C

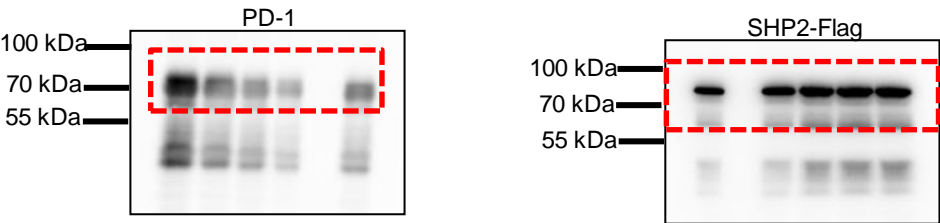

Figure 3E

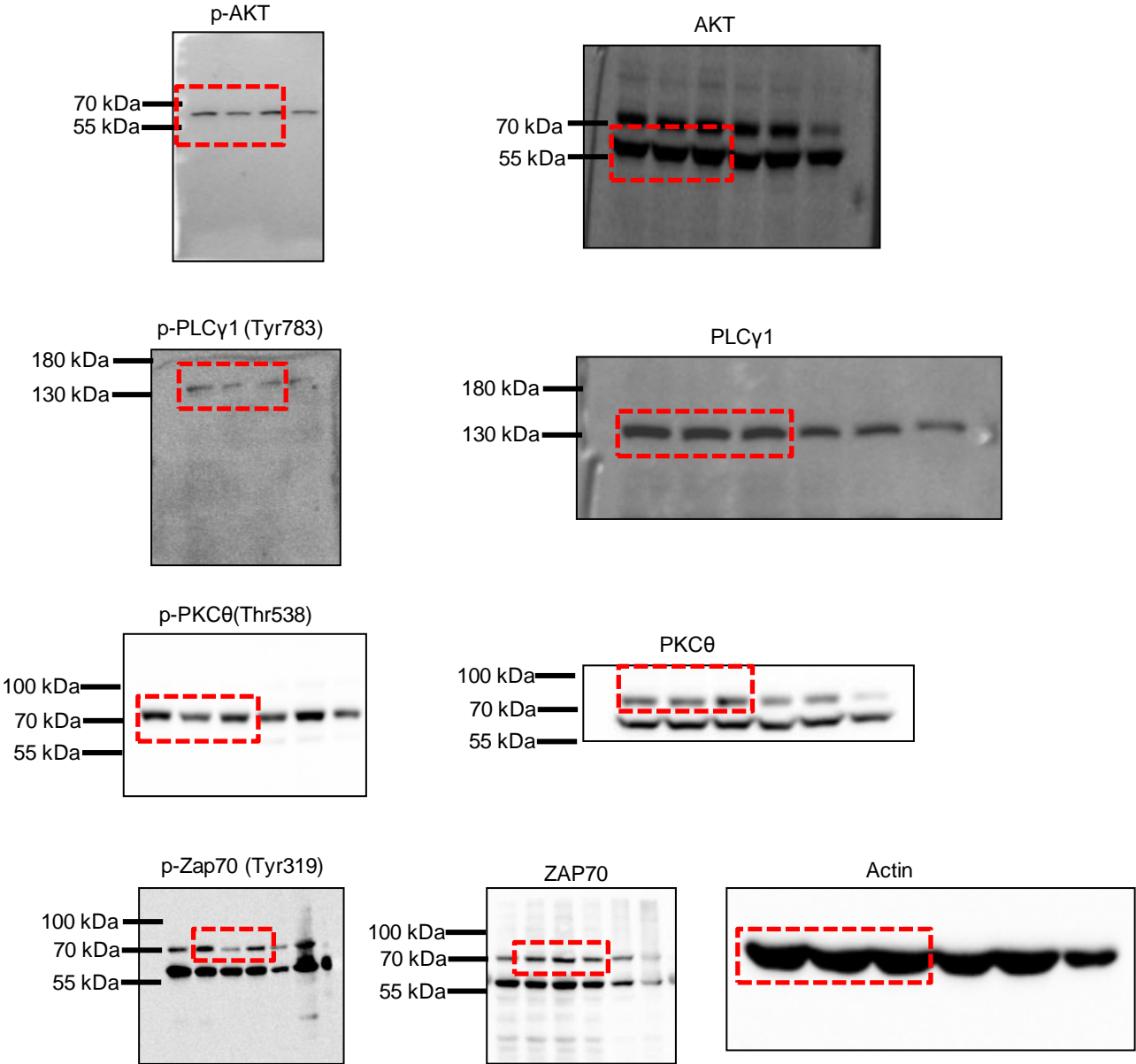

Figure 3F

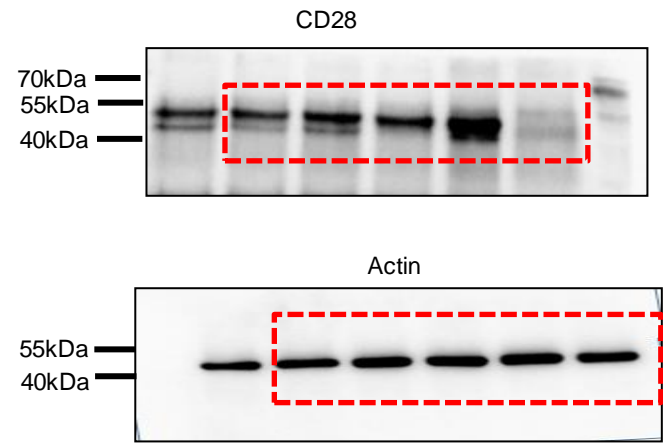

Figure 3G

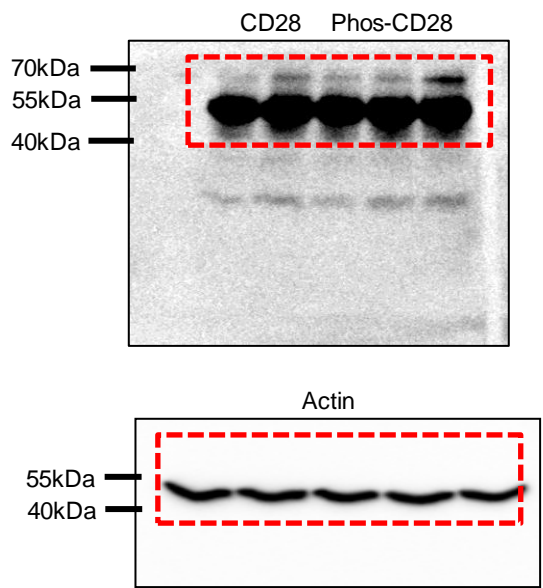

Supplement: Supplementary file 5 — Source Data for Figure 3 [file EMMM-12-e11571-s003.pdf]
